# Precise Modulation of Reorganization Energy through Methyl Substitution for High Performance Organic Solar Cells

**DOI:** 10.1002/advs.202505143

**Published:** 2025-07-07

**Authors:** Li Chen, Chaoyue Zhao, Joshua Yuk Lin Lai, Rongkun Zhou, Aleksandr Sergeev, Kam Sing Wong, Huawei Hu, Zilong Zheng, Han Yu, Sai Ho Pun, Guangye Zhang, He Yan

**Affiliations:** ^1^ Department of Chemistry and Hong Kong Branch of Chinese National Engineering Research Center for Tissue Restoration and Reconstruction The Hong Kong University of Science and Technology Hong Kong 999077 P. R. China; ^2^ College of Materials Science and Engineering Beijing University of Technology Beijing 100124 P. R. China; ^3^ Department of Physics and William Mong Institute of Nano Science and Technology Hong Kong University of Science and Technology Clear Water Bay Kowloon Hong Kong 999077 China; ^4^ State Key Laboratory for Modification of Chemical Fibers and Polymer Materials College of Materials Science and Engineering Donghua University Shanghai 201620 China; ^5^ College of New Materials and New Energies Shenzhen Technology University Shenzhen 518118 P. R. China

**Keywords:** methyl substitution, organic solar cells, quinoxaline, reorganization energy, small molecule acceptor

## Abstract

Efficient charge transport and minimized energy loss are critical for advancing the performance of organic solar cells (OSCs). In this study, a series of quinoxaline‐based electron acceptors, BQx‐MeF, BQx‐MeCl, and BQx‐MeBr, featuring methyl and halogen substitutions is designed and synthesized to systematically modulate reorganization energy (λ) and film morphology. Quantum chemical calculations confirmed that methylation effectively reduces λ by limiting structural relaxation, leading to suppressed non‐radiative recombination energy loss (Δ*E*_nr_) and improved charge transport. Among the synthesized materials, BQx‐MeCl exhibited the lowest energy loss and the most balanced electron and hole mobilities, resulting in a superior power conversion efficiency (PCE) of 19.2% in a binary device. In optimized ternary OSCs, BQx‐MeCl further reached a remarkable PCE of 19.6%. This enhancement is attributed to optimized molecular stacking, improved film morphology, and reduced trap‐assisted recombination. These findings highlight the pivotal role of molecular design in lowering reorganization energy to minimize energy losses and maximize charge collection, offering an effective strategy for the development of high‐efficiency OSCs.

## Graphical abstract

This study reports the design of quinoxaline‐based electron acceptors with tailored methyl and halogen substitutions to modulate reorganization energy (λ) and enhance charge transport. we demonstrated that methylation effectively reduces λ, leading to minimized energy losses and improved device performance. The resulting acceptor, BQx‐MeCl, achieves a power conversion efficiency of 19.2% in binary devices and 19.6% in optimized ternary devices.

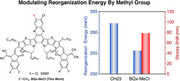

## 1 Introduction

Understanding and mitigating energy loss (*E*_loss_) is pivotal for advancing the performance of organic solar cells (OSCs),^[^ ^1^ ^]^ which hold immense potential for revolutionizing renewable energy with lightweight, flexible, and scalable solar technologies. However, OSCs still lag behind silicon and perovskite solar cells in performance,^[^ ^2^ ^]^ primarily due to relatively low open‐circuit voltage (*V*_OC_) and substantial energy losses,^[^ ^3^ ^]^ especially from non‐radiative recombination energy loss (Δ*E*_nr_).^[^ ^4^ ^]^ Additionally, challenges in morphological control, particularly achieving optimal miscibility between donor and acceptor materials, hinder further efficiency improvements.^[^ ^5^ ^]^ Addressing these issues through molecular design focuses on synthesizing molecules that minimize Δ*E*_nr_ and enhance morphology, thereby pushing OSC efficiency beyond current benchmarks.

The primary mechanism behind non‐radiative recombination energy loss stems from exciton‐vibration coupling, where vibrational relaxation facilitates the decay of excited states to the ground state.^[^ ^6^ ^]^ This coupling is influenced by the reorganization energy (λ), a parameter reflecting the structural relaxation required during electronic transitions. Lowering λ is essential for suppressing exciton‐vibration coupling, thus reducing energy losses and improving exciton lifetimes, diffusion lengths, charge transport, and mobility.^[^ ^6b^ ^]^ Furthermore, according to the classical Marcus theory of electron transfer: $k_{et}=\frac{2\pi }{\hbar }H^{2}\frac{1}{\sqrt{4\pi \lambda k_{B}T}}\;\exp\;\left(-\frac{{\left(\Delta G+\lambda \right)}^{2}}{4\lambda k_{B}T}\right)$, where *H* is the electronic coupling term between initial and final states, Δ*G* is the Gibbs free energy change, *k*_B_T is the thermal energy, and λ is the reorganization energy.^[^ ^5^, ^7^ ^]^ A lower λ reduces the energetic barrier for exciton dissociation and promotes efficient charge transfer, ultimately enhancing device performance.

In this study, we introduce methyl groups to the quinoxaline core of Y‐series electron acceptors to modulate λ and improve morphology. Methylation, commonly employed to influence solubility and electronic properties in organic semiconductors,^[^ ^8^ ^]^ can also effectively reduce λ by diminishing electronic‐vibrational coupling through limiting structural relaxation and enhancing molecular packing. This strategy offers a straightforward and chemically versatile alternative to more complex molecular design approaches.

**Figure 1:**
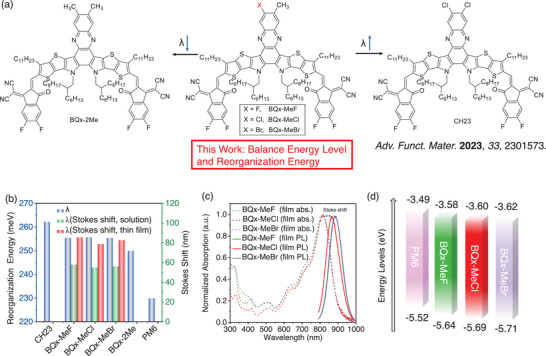
a) Molecular design strategies of methyl substitution on the central core of Quinoxaline (Qx) and the chemical structures of electron acceptors in this work. b) A summary of the reorganization energy (blue) of electron acceptors and donor PM6; the Stokes shift of the three electron acceptors both in thin film (red) and chloroform solution (green). c) Normalized ultraviolet‐visible absorption spectra of the three electron acceptors as thin film, in comparison with their photoluminescence spectra (with the Stokes shift denoted). d) Energy level diagram of the related materials.

Guided by quantum chemical calculations, we designed and synthesized three quinoxaline‐based electron acceptors with different halogen substitutions: BQx‐MeCl, BQx‐MeF, and BQx‐MeBr (**Figure** **1**a). Starting with a quinoxaline core substituted with two chlorine atoms (BQx‐Cl_2_, named CH23),^[^ ^9^ ^]^ we calculated *λ* and compared it with cores featuring one methyl and one halogen substituent (BQx‐MeCl, BQx‐MeF, BQx‐MeBr) and two methyl groups (BQx‐Me_2_). As shown in Figure 1b, increasing methyl substitution significantly reduced λ, bringing it closer to that of PM6, a widely used donor material. However, introducing two methyl groups (BQx‐Me_2_) elevated the highest occupied molecular orbital (HOMO) energy level, resulting in an unfavorable energy level mismatch with PM6. In contrast, BQx‐MeCl achieved an optimal balance by reducing *λ* while maintaining energy level compatibility and enhancing morphology. Among the synthesized acceptors, BQx‐MeCl exhibited the most promising performance. PM6:BQx‐MeCl‐based devices achieved a remarkable PCE of 19.2%, driven by a significant reduction in Δ*E*_loss_ and enhanced *V*_OC_. The introduction of the methyl group not only lowered λ but also improved molecular packing, facilitating better charge transport, morphological stability, and miscibility. This study highlights methylation as an innovative and scalable strategy for improving OSC performance. By leveraging DFT calculations and focusing on reorganization energy, we offer a new design paradigm for high‐efficiency organic photovoltaics. This approach provides a practical pathway to address energy loss and accelerate OSC commercialization.

## 2 Results and Discussion

The synthetic routes for the three Qx‐based acceptors, BQx‐MeF, BQx‐MeCl, and BQx‐MeBr, are depicted in Scheme S1 (Supporting Information) and closely follow the synthetic methodologies we previously reported in the literature.^[^ ^10^ ^]^ Comprehensive synthetic methodologies and characterization techniques, including ¹H NMR, ¹^3^C NMR, and mass spectrometry, are detailed in the Figures S13–S39 (Supporting Information). Thermogravimetric analysis (TGA) conducted under a nitrogen atmosphere demonstrated the excellent thermal stability of BQx‐MeF, BQx‐MeCl, and BQx‐MeBr, with a mass loss of 5% recorded at 323.8, 325.6, and 325.3 °C, respectively (Figure S1a, Supporting Information).

The ultraviolet‐visible (UV–vis) absorption spectra of BQx‐MeF, BQx‐MeCl, and BQx‐MeBr in chloroform (CF) solution and thin films were investigated (Figure 1c; Figure S1b,c, Supporting Information), with detailed optical properties summarized in Table S1 (Supporting Information). These three acceptors exhibited similar absorption spectra in both solution and film states due to their identical conjugated frameworks. As shown in Figure 1c, the maximum absorption peaks (*λ*_max, sol_) gradually blue‐shifted from BQx‐MeF, BQx‐MeCl, to BQx‐MeBr with absorption peaks of 756, 754, and 751 nm, respectively. This is attributed to the increased electron‐withdrawing nature of the central atom, resulting in weaker intramolecular charge transfer effects. Photoluminescence (PL) spectra in both thin film and chloroform solution were also measured (Figure 1c; Figure S1b,c,e, Supporting Information). Notably, the Stokes shifts in thin films for BQx‐MeF, BQx‐MeCl, and BQx‐MeBr are 86, 79, and 83 nm, respectively (Figure 1b,c), which is significantly smaller than that of CH23 (≈120 nm). This reduction suggests that methyl substitution effectively suppresses molecular vibration, which is in good agreement with DFT calculations showing that the introduction of a methyl group leads to reduced reorganization energy. Cyclic voltammetry (CV) measurements determined the HOMO and LUMO energy levels of the three acceptors (Figure 1d; Figure S1f, Supporting Information). BQx‐MeF, BQx‐MeCl, and BQx‐Br exhibit HOMO/LUMO levels of −5.64/−3.58, −5.69/−3.60, and −5.71/−3.62 eV (Table S1, Supporting Information and Figure 1d), respectively. Density functional theory (DFT) calculations were performed to investigate the influence of halogen substituents and the methyl group on the frontier molecular orbitals of these small molecular acceptors (SMAs). The calculated LUMO/HOMO energy levels (Figure S2a–c, Supporting Information) align well with the results obtained from CV measurements.

**Figure 2:**
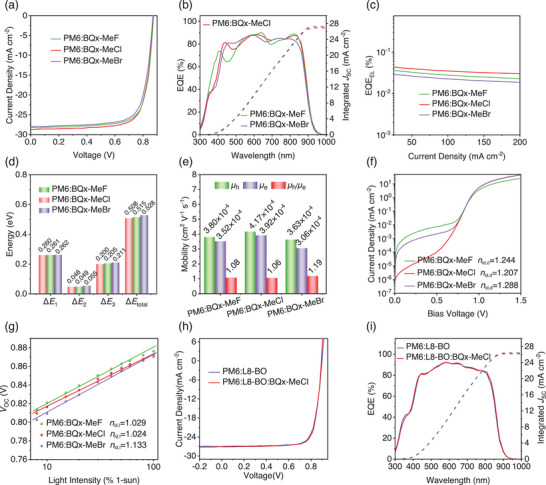
a) Current density‐voltage (*J*–*V*) curves of the BQx‐MeF, BQx‐MeCl, and BQx‐MeBr‐based optimized devices. b) EQE spectra and integrated *J*_SC_s of the optimized devices. c) EQE_EL_ f the PM6:SMA based OSCs. d) Detailed *E*_loss_ of BQx‐MeF, BQx‐MeCl, and BQx‐MeBr based devices. e) Histograms of the *µ*_h_ and *µ*_e_ of the OSCs based on the PM6:BQx‐MeF, PM6:BQx‐MeCl, and PM6:BQx‐MeBr. f) *V*_OC_ versus light intensity. g) Dark *J*–*V* curves. h) Current density‐voltage (*J*–*V*) of the BQx‐MeF, BQx‐MeCl, and BQx‐MeBr‐based ternary devices. i) EQE spectra of the optimized ternary devices.

Devices based on BQx‐MeF, BQx‐MeCl, and BQx‐MeBr were fabricated using the conventional device architecture: ITO/PEDOT:PSS/PM6:acceptors/PNDIT‐F3N/Ag, to evaluate their photovoltaic performance. 1,3,5‐Trichlorobenzene (TCB) was added as a solvent additive in chloroform solutions to optimize the photovoltaic performance of the OSCs. Device optimization for different spin‐coating speeds and is shown in Figure S3 and Table S2 (Supporting Information) of support information. Detailed device optimization procedures are provided in the Supporting Information. The current density‐voltage (*J*–*V*) curves of the optimized devices are shown in **Figure** **2**a, and their corresponding photovoltaic parameters are summarized in **Table** **1**. The BQx‐MeF and BQx‐MeBr‐based devices achieved PCEs of 18.8% and 18.4%, with open‐circuit voltages (*V*_OC_) of 0.878 and 0.872 V, short‐circuit currents (*J*_SC_) of 28.21 and 28.01 mA cm⁻^2^, and fill factors (FF) of 76.1% and 75.3%, respectively (Table 1). Notably, the BQx‐MeCl‐based OSC exhibited a remarkable PCE of 19.2%, with a *V*_OC_ of 0.876 V, *J*_SC_ of 28.71 mA cm⁻^2^, and FF of 76.1%. The external quantum efficiency (EQE) spectra of the OSCs, shown in Figure 2b, verify the reliability of the *J*_SC_ values. All devices exhibited broad charge generation from 300 to 920 nm due to the complementary absorption profiles of the polymer donor (PM6) and the acceptors. The denser crystal packing of BQx‐MeCl, relative to BQx‐MeF and BQx‐MeBr, contributed to the wider EQE spectral response in devices based on BQx‐MeCl. Furthermore, the inclusion of TCB influenced the aggregation behavior of BQx‐MeCl. Variations in EQE response among the devices can be ascribed to differences in active layer morphology. Notably, the integrated *J*_SC_ value obtained from the EQE spectrum for PM6:BQx‐MeCl was 27.31 mA cm⁻^2^ (Table 1), which closely aligns with the device results. The improved PCE and FF for the BQx‐MeCl‐based devices indicate more efficient charge transport and reduced charge recombination. Moreover, the photoluminescence (PL) quenching results of the three blends (Figure S1d,e, Supporting Information) show a similar trend. When excited at 514/785nm, the PL quenching efficiencies were found to be 91.4%/91.5% for PM6:BQx‐MeF, 96.1%/97.3% for PM6:BQx‐MeCl, and 86.7%/86.5% for PM6:BQx‐MeBr, respectively. The higher quenching efficiencies observed in the PM6 blends suggest more effective electron and hole transfer processes, leading to the best (EQE) response and the highest (*J*_SC_) among the three devices.

**Table 1:** Summary of device parameters for optimized OSCs.

Active layer	*V*_OC_ [V]	*J*_SC_ [mA cm⁻^2^]	*J*_SC_,_cal_ ^a)^ [mA cm⁻^2^]	FF [%]	PCE^b)^ [%]
PM6:BQx‐MeF	0.878 (0.880 ± 0.004)	28.21 (28.19 ± 0.03)	27.01	76.1 (75.4 ± 0.007)	18.8 (18.7 ± 0.2)
PM6:BQx‐MeCl	0.876 (0.877 ± 0.002)	28.71 (28.65 ± 0.06)	27.31	76.1 (76.4 ± 0.004)	19.2 (19.1 ± 0.2)
PM6:BQx‐MeBr	0.872 (0.871 ± 0.001)	28.01 (28.01 ± 0.05)	26.93	75.3 (75.0 ± 0.002)	18.4 (18.3 ± 0.1)
PM6:L8‐BO	0.900 (0.900 ± 0.001)	27.03 (26.75 ± 0.32)	26.20	78.4 (77.7 ± 0.007)	19.0 (18.8 ± 0.2)
PM6:L8‐BO:BQx‐MeCl	0.894 (0.894 ± 0.001)	27.54 (27.15 ± 0.21)	26.40	79.6 (79.4 ± 0.002)	19.6 (19.4 ± 0.2)

^a)^ Statistical and optimal results are listed in parentheses and outside of parentheses, respectively. The average parameters were calculated from 10 independent devices;

^b)^ Current densities calculated from EQE curves.

To understand the effects of halogen and methyl group modifications on energy loss in OSCs related to the central unit of the three acceptors, a detailed energy loss (*E*_loss_) analysis^[^ ^11^ ^]^ was conducted, with the methods outlined in the supporting information (Figure S4, Supporting Information). The data, presented in Figure 2c,d and Table S3 (Supporting Information), For the Δ*E*_1_, show that all devices had similar Δ*E*_1_ values of ≈0.260 eV, indicating minimal contribution to *V*_OC_ differences. However, Δ*E*_2_ values of 0.049 eV for BQx‐MeF, 0.048 eV for BQx‐MeCl, and 0.055 eV for BQx‐MeBr point to charge‐transfer state losses as a factor for the *V*_OC_ variance. The most significant contributor, Δ*E*_3_, reflects nonradiative recombination, with values of 0.200, 0.205, and 0.211 eV for BQx‐MeF, BQx‐MeCl, and BQx‐Br devices, respectively, which are superior than that of CH23 (0.237 eV),^[^ ^9^ ^]^ which indicated the introduction of methyl and halogen substituents to the quinoxaline (Qx) core in electron acceptors effectively reduces reorganization energy. Optical bandgaps (*E*_g_) were determined to be 1.394, 1.396, and 1.404 eV, corresponding to calculated *E*_loss_ values of 0.515, 0.508, and 0.528 eV for BQx‐MeF, BQx‐MeCl, and BQx‐Br devices, respectively (Table S3, Supporting Information). The lower *E*_loss_ values in these three systems contribute to their higher *V*_OC_. The higher electroluminescence quantum efficiency (EQE_EL_) values for BQx‐MeCl (4.3 × 10^−4^) compared to BQx‐MeF (3.6 × 10^−4^) and BQx‐MeBr (2.8 × 10^−4^) (Figure 2c) further confirm its enhanced molecular arrangement and reduced energy losses (Figure 2d; and Table S3, Supporting Information). This result confirms the design rationale, demonstrating that the molecular strategy effectively reduces reorganization energy, achieves lower energy loss and higher PCE.

The suppressed bimolecular recombination and efficient charge collection are intricately linked to the mobility of the materials. The charge transport properties and recombination mechanisms were probed through space‐charge‐limited current (SCLC) measurements,^[^ ^12^ ^]^ revealing hole and electron mobilities (*µ*_h_/*µ*_e_) of 3.8 × 10^−4^ / 3.52 × 10^−4^ cm^2^ V⁻¹ s⁻¹ for PM6:BQx‐MeF, 4.17 × 10^−4^ / 3.92 × 10^−4^ cm^2^ V⁻¹ s⁻¹ for PM6:BQx‐MeCl, and 3.63 × 10^−4^ / 3.06 × 10^−4^ cm^2^ V⁻¹ s⁻¹ for PM6:BQx‐MeBr devices (Figure 2e; Figure S5 and Table S4, Supporting Information). The balanced carrier transport in BQx‐MeCl (*µ*_h_/*µ*_e_ ratio of 1.06) contributes to the higher *J*_SC_ and FF values in these OSCs. To assess the charge recombination behavior in OSCs, the relationship between *J*_SC_, *V*_OC_, and light intensity (*P*_light_) was examined.^[^ ^13^ ^]^ The recombination parameters (S) for devices based on BQx‐MeF, BQx‐MeCl, and BQx‐MeBr were found to be 0.9767, 0.9775, and 0.9702, respectively (Figure S6a and Table S4, Supporting Information). These similar S values suggest that the degree of bimolecular recombination in these devices is comparably low. To further investigate trap‐assisted recombination,^[^ ^10^, ^14^ ^]^ we measured the dark ideality factor (*n*_id,d_) for each device. A value close to 1 indicates minimal trap‐assisted recombination, while higher values suggest increased recombination due to traps. The dark *J*–*V* curves are presented in Figure 2f. The *n*_id,d_ for the OSC based on BQx‐MeCl was 1.207, lower than 1.244 for PM6:BQx‐MeF and 1.288 for PM6:BQx‐MeBr (Figure 2f; and Table S4, Supporting Information), indicating reduced trap‐assisted recombination in the BQx‐MeCl device. In addition, the ideality factor (*n*_id,l_) can also be calculated by analyzing the relationship between the *V*_OC_ of the device and light intensity. The corresponding *V*_OC_ – *I* curves are shown in Figure 2g, and the resultant *n*_id,l_ values of 1.029, 1.024, and 1.133 (Table S4, Supporting Information), for PM6:BQx‐MeF, PM6:BQx‐MeCl, and PM6:BQx‐MeBr, respectively, were obtained. The device based on PM6:BQx‐MeCl exhibited the lowest *n*_id,l_ value, suggesting effective suppression of trap‐assisted recombination, which ensures efficient charge generation and transport. The PM6:BQx‐MeCl device demonstrates both weak bimolecular recombination and reduced trap‐assisted recombination, consistent with the observed trends in device mobility and FF.

To investigate the charge carrier generation and transport behaviors in devices utilizing these acceptors under operational conditions using the photoinduced charge extraction by linearly increasing voltage (photo‐CELIV) technique,^[^ ^15^ ^]^ as shown in Figure S6b (Supporting Information). The devices incorporating BQx‐MeCl exhibited a higher average charge mobility (*µ*_avg_) of 1.52 × 10^−4^ cm^2^ V^−^¹ s^−^¹, surpassing the values of 1.38 × 10^−4^ cm^2^ V^−^¹ s^−^¹ for BQx‐MeF and 1.27 × 10^−4^ cm^2^ V^−^¹ s^−^¹ for BQx‐MeBr. This alignment with the SCLC results confirms the superior carrier mobility of BQx‐MeCl. Additionally, we assessed the influence of halogen and methyl substitutions on charge transport properties using impedance spectroscopy (IS) (Figure S6c, Supporting Information).^[^ ^16^ ^]^ The BQx‐MeCl‐treated organic solar cells exhibited the lowest resistances compared to the other devices, suggesting effective donor‐acceptor phase separation in bulk heterojunctions (BHJs) and enhanced interfacial contact between the layers. Transient photocurrent (TPC) (Figure S6d, Supporting Information) and transient photovoltage (TPV) (Figure S6e, Supporting Information) measurements revealed the charge recombination characteristics. BQx‐MeCl exhibited shorter TPC decay times (*τ*_TPC_ = 0.269 µs) and extended TPV decay times (*τ*_TPV_ = 3.30 µs), confirming fewer recombination and more efficient charge extraction. The above tests indicate that the superior performance of BQx‐MeCl can be primarily attributed to its more balanced electron/hole transport, improved crystallinity, and optimized miscibility, which collectively enhance molecular stacking and morphology of the active layer. Unlike BQx‐MeF and BQx‐MeBr, BQx‐MeCl achieves the best performance due to its optimal combination of electronic effects and structural organization. The methyl group acts as a weak electron donor, while the chlorine atom serves as an electron‐withdrawing group, subtly modulating the energy levels and intermolecular interactions. This molecular design effectively minimizes recombination losses and enhances charge transport, leading to superior OSC performance.

To further explore the photovoltaic performance of the BQx‐MeCl as a guest acceptor, we fabricated ternary devices with the configuration ITO/PEDOT/PM6:L8‐BO/PNDIT‐F3N/Ag. The optimal *J*–*V* and EQE characteristics for the ternary system are presented in Figure 2h,i, respectively, with the corresponding device parameters detailed in Table 1. The control binary device utilizing PM6:L8‐BO achieved a PCE of 19.0%. In contrast, the ternary device PM6:L8‐BO:BQx‐MeCl exhibited a significant enhancement in FF, reaching 79.59%, culminating in an impressive PCE of 19.60%. These findings highlight the promising role of the BQx‐MeCl can adjust morphology to obtain great photovoltaic performance of both binary and ternary systems.

In‐situ UV‐Vis absorption measurements were performed to investigate the film formation process of PM6:acceptors, revealing three distinct stages of film formation.^[^ ^17^ ^]^ During solvent evaporation, the absorption peak positions remained stable in the initial stage. As the system approached supersaturation, a clear red‐shift in absorption peaks was observed due to molecular aggregation. PM6:BQx‐MeCl exhibited faster film formation (0.225 s) compared to PM6:BQx‐MeF (0.255 s) and PM6:BQx‐MeBr (0.276 s) (Figure S7a–f, Supporting Information). The rapid crystallization of BQx‐MeCl promotes more favorable phase separation, leading to enhanced exciton dissociation and carrier transport. These results are in good agreement with the higher PCE observed in BQx‐MeCl devices.

**Figure 3:**
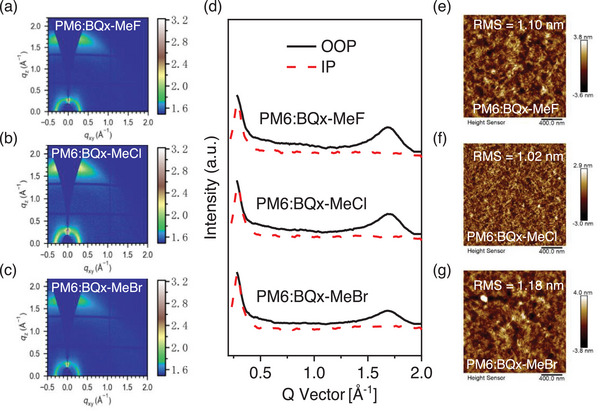
Morphology characterization of the blend films. a–c) 2D GIWAXS patterns. and (d) 1D line‐cuts of the PM6:BQx‐MeF, PM6:BQx‐MeCl and PM6:BQx‐MeBr. e,f) AFM height images.

These acceptors, featuring distinct molecular configurations depending on the core, influence intermolecular packing and film crystallinity. To explore the relationship between molecular structure and device performance, grazing‐incidence wide‐angle X‐ray scattering (GIWAXS) was employed to study the film morphology.^[^ ^18^ ^]^ Figures S8a–d (Supporting Information) and **Figure** **3**a–d present the 2D GIWAXS patterns and corresponding line‐cut curves for both the pure and blend acceptor films. All three acceptors (pure and blend films) show distinct (100) diffraction peaks in the in‐plane (IP) direction, with lamellar stacking distances ranging from 0.29‐0.30 Å (Table S5, Supporting Information), and prominent (010) diffraction peaks in the out‐of‐plane (OOP) direction, with *π*–*π* stacking distances of 1.72–1.73 Å (Table S5, Supporting Information), with packing distances ranging from 3.64 to 3.65 Å (Table S5, Supporting Information). These findings suggest good molecular crystallinity and a favorable face‐on orientation in molecular packing. Notably, the BQx‐MeCl‐based films exhibit the largest crystal coherence lengths (CCLs) in OOP and IP directions (Table S5, Supporting Information), indicating more ordered molecular packing and stronger crystallinity compared to the other two systems. Therefore, adding methyl and chlorine‐substituents to the Qx core can enhance the crystallization of SMAs, improving charge transport for increased *J*_SC_s and FFs in OSCs. Further analysis of the blend film morphology through atomic force microscopy (AFM) (Figure 3e–g; Figure S8e–g, Supporting Information) reveals fibrillar network structures across all blend films, facilitating charge transfer. The root‐mean‐square (RMS) roughness of PM6:BQx‐MeCl, measured at 1.02 nm, is slightly lower than that of PM6:BQx‐MeF (1.10 nm) and PM6:BQx‐MeBr (1.18 nm), indicating enhanced compatibility between the donor and acceptor in the BQx‐MeCl system. Additionally, we also tested the morphology of these SMAs. AFM analysis showed that different solubilities correspond to different roughness levels. Among them, the morphology of the three molecules treated with chloroform is good, while the morphology treated with toluene is poor. (Figure S8h–s, Supporting Information). The ordered molecular packing and network morphology in BQx‐MeCl‐based devices contribute to reduced charge recombination and enhanced PCE.

**Figure 4:**
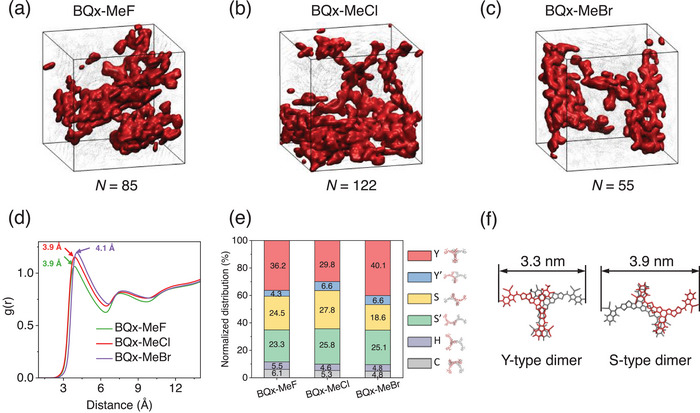
Impact of stacking mode distributions of pure films. The maximum conductive networks (marked in red) in the acceptor films of a) BQx‐MeF; b) BQx‐MeCl; c) BQx‐MeBr from MD simulations, with the assembled molecule number (*N*) shown below. d) Radial distribution function g(r) of acceptor:acceptor backbones stacking in the three pure acceptor films. e) The distributions of five dimer stacking structures, (C‐, S‐, S’‐, Y’‐, and Y‐type) of three acceptors. f) Spatial extension of Y‐type and S‐type aggregates, S‐type exhibits a better spatial extension than Y‐type.

Molecular Dynamic (MD) simulations were employed to gain deeper insights into the molecular interaction within the three systems.^[^ ^5^, ^19^ ^]^ (**Figure** **4**a–f; Figure S9, Supporting Information) The continuity of the conductive network of the simulated molecular packing, illustrated in Figure 4a–c, reveals that BQx‐MeCl tend to form more compact and homogeneous thin films, indicating stronger intermolecular interaction. MD simulation data were analyzed to obtain the radial distribution function (RDF), which describes the density of surrounding matter as a function of distance from a point. The RDF plot for BQx‐MeF, BQx‐MeCl, and BQx‐MeBr systems is presented in Figure 4d. The position of the first peak indicates the average molecular stacking distance, while the peak height reflects the local structural order, with higher peaks signifying greater molecular aggregation. As the substituent atoms change from F to Cl and Br, the peak height increases, primarily due to the decreasing electronegativity of the substituents, which reduces the electric dipole moment of the SMAs and facilitates their aggregation in the CF. Although the enhanced conjugated backbone stacking promotes the formation of long‐range conductive networks, the larger atomic radius of Br increases the molecular stacking distance (from 3.9 Å for BQx‐MeF and BQx‐MeCl to 4.1 Å for BQx‐MeBr), ultimately reducing the extensibility of the conductive network in BQx‐MeBr films. Furthermore, Figure 4e shows the effect of halogens on the central core quinoxaline (Qx) of these three acceptors on the proportions of the five stacking modes. The substitution of halogen atoms on the Qx core primarily affects the proportions of Y‐ and S‐type packing, which in turn significantly influences the extensibility of the conductive network. Figure 4f illustrates that the S‐type dimer exhibits greater spatial extension (3.9 nm) compared to the Y‐type dimer (3.3 nm). A higher content of dimers with better spatial extension is more favorable for the formation of long‐range conductive networks. Among the three systems, BQx‐MeCl has the highest content of S‐type packing and the lowest content of Y‐type packing, resulting in the highest electron mobility achieved. Overall, this result is in good agreement with the enhanced thin film crystallinity observed in GIWAXS results. The morphological study suggests that the BQx‐MeCl based film exhibits tighter molecular interactions and stronger *π*–*π* stacking, followed by BQx‐MeF and BQx‐MeBr. The increased crystallinity in OOP direction contributes to the formation of a robust transport channel, thereby enhancing electron mobility, consistent with SCLC test results.

**Figure 5:**
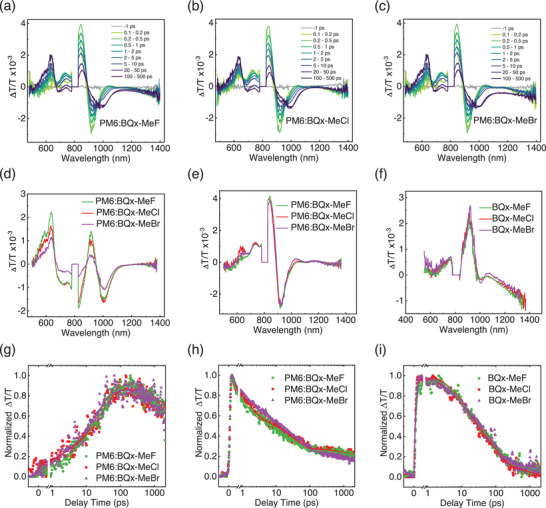
Exciton and charge dynamics. Transient absorption spectra of a) PM6:BQx‐MeF, b) PM6:BQx‐MeCl, and c) PM6:BQx‐MeBr under 800 nm excitation. Spectral components of decomposed transient absorption spectra illustrating d) donor GSB together with hole polaron, e) local excited state of acceptor, and f) delocalized state of pure acceptor as well as g–i) their corresponding kinetics.

To investigate charge transfer processes in blend films, transient absorption (TA) spectroscopy^[^ ^20^ ^]^was conducted under 800 nm photoexcitation (**Figure** **5**; Figures S10–S12 and Tables S6–S8, Supporting Information). The TA spectra were decomposed using the multivariate curve resolution‐alternating least square (MCR‐ALS) method, revealing three main time and spectral components. Component I, attributed to the ground state bleaching (GSB) of the donor (PM6) and hole polaron signal due to hole transfer from the acceptor (these three SMAs) to the donor (Figure 5d). We obtained the exciton dissociation and diffusion times (Figure 5g; and Table S6, Supporting Information) through biexponential fitting of the hole transfer kinetics, indicating that the PM6:BQx‐MeCl exhibited good interfacial exciton diffusion and dissociation, which is beneficial for the generation of free charges. Component II represented the local excited state of the acceptor (Figure 5e,h; and Table S7, Supporting Information). Fast decay for BQx‐MeCl‐based device indicate fast exciton delocalization, which is consistent with the fastest building‐up of delocalized state (component III, Figure 5f,i; and Table S8, Supporting Information). The fastest delocalization time for BQx‐MeCl suggests efficient exciton dissociation and free carrier formation in BQx‐MeCl device. To sum up, the fastest exciton diffusion time (*τ*_2_ component of hole transfer kinetics) of PM6:BQx‐MeBr device together with the lowest intensities of donor GSB and hole polaron bands, and fastest quenching of local exciton state are likely due to non‐radiative losses of photogerated carriers, which may explain the low PCE of PM6:BQx‐MeBr‐based OSCs. At the same time, the fastest exciton dissociation time for PM6:BQx‐MeCl, deduced from *τ*_1_ component of hole transfer kinetics, together with the highest intensity of hole polaron band ≈1000 nm, and fastest building‐up of delocalized state are well‐consistent with its highest PCE. Thus, the BQx‐MeCl device demonstrated a balanced trade‐off between improved charge carrier generation, recombination lifetimes, and carrier transport properties, resulting in enhanced device performance.

## 3 Conclusion

In conclusion, the introduction of methyl and halogen substituents to the quinoxaline (Qx) core in electron acceptors effectively reduces reorganization energy and optimizes morphological properties, leading to enhanced OSC performance. Among the synthesized SMAs, BQx‐MeCl demonstrated superior device efficiency with a PCE of 19.2% in binary OSCs and 19.6% in ternary OSCs, outperforming BQx‐MeF and BQx‐MeBr due to its balanced carrier mobilities, reduced non‐radiative recombination, and improved morphology. This study underscores the critical role of reorganization energy modulation in minimizing energy loss and optimizing charge transport, providing a valuable design strategy for next‐generation high‐efficiency organic solar cells.

## Conflict of Interest

The authors declare no conflict of interest.

## Supporting information



Supporting Information

Supporting Information

## Data Availability

Research data are not shared.
