# Supplementary material for: Precise Modulation of Reorganization Energy through Methyl Substitution for High Performance Organic Solar Cells
Source: Adv Sci (Weinh). 2025 Jul 7;12(31):e05143. doi: 10.1002/advs.202505143 (PMC12376521; doi:10.1002/advs.202505143)
Supplement: Supplementary file 1 — Supporting Information [file ADVS-12-e05143-s001.docx]

## Supporting information for

**Precise Modulation of Reorganization Energy through Methyl Substitution for High Performance Organic Solar Cells**

Li Chen, Chaoyue Zhao, Joshua Yuk Lin Lai, Rongkun Zhou, Aleksandr Sergeev, Kam Sing Wong, Huawei Hu, Zilong Zheng, Han Yu,^*^ Sai Ho Pun,^*^ Guangye Zhang,^*^ He Yan^*^

L. Chen, C. Zhao, J. Y. L. Lai, H. Yu, S. H. Pun, H. Yan

Department of Chemistry and Hong Kong Branch of Chinese National Engineering Research Center for Tissue Restoration and Reconstruction
The Hong Kong University of Science and Technology 999077 Hong Kong China
E-mail: yuhan.yu@polyu.edu.hk; punsaiho@ust.hk; hyan@ust.hk

G. Zhang

College of New Materials and New Energies, Shenzhen Technology University,
Shenzhen 518118, China
E-mail: zhangguangye@sztu.edu.cn

R. Zhou, Z. Zheng

College of Materials Science and Engineering
Beijing University of Technology
Beijing,100124, P.R. China

A. Sergeev, K. S. Wong

Department of Physics and William Mong Institute of Nano Science and Technology
Hong Kong University of Science and Technology
Clear Water Bay, Kowloon 999077, Hong Kong, China

H. Hu

State Key Laboratory for Modification of Chemical Fibers and Polymer Materials
College of Materials Science and Engineering
Donghua University, Shanghai 201620, China

**Table of Contents**

1. Materials and Methods

2. Synthesis of BQx-MeF, BQx-MeCl and BQx-MeBr

3. Supplementary Figures

4. Supplementary Tables

5. References

**1. Materials and Methods**

**Materials.**

Toluene was refluxed and distilled from sodium with benzophenone as an indicator before use. N, N'-Dimethylformamide (DMF), n-Butyllithium (n-BuLi, 1.6 M in THF), and Pd_2_(dba)_3_ were purchased from Alfa Aesar or J&K Chemical or Derthon (Shenzhen China) Optoelectronic Materials Science Technology Co., Ltd. The synthetic routes to the three BQx-based SMAs were outlined in Scheme S1. Synthetic details of BQx-based compounds and target SMAs were showed as follows. All other reagents and chemicals were purchased from commercial sources and used without further purification^1^H and ^13^C NMR spectra were obtained with a Bruker AV-400 MHz NMR spectrometer. Chemical shifts were reported in parts per million (ppm, *δ*)^1^H, and^13^C NMR spectra were referenced to tetramethylsilane (0 ppm) in CDCl_3_. Mass spectra were collected on a MALDI Micro MX mass spectrometer, or an API QSTAR XL System.

**Device fabrication procedure.**

Device Fabrication and Testing The OSCs with conventional structure were fabricated on glass/ITO (indium tin oxide)/PEDOT:PSS (poly(3,4-ethylenedioxythiophene):poly(styrene sulfonate))/active layer/PNDIT-F3N ([(9,9-bis(3'-(N,N-dimethylamino)propyl)-2,7-fluorene)-alt-5,5'-bis(2,2'-thiophene)-2, 6-naphthalene-1,4,5,8-tetracaboxylic-N,N'-di(2-ethylhexyl)imide])/Ag. The patterned ITO-coated glass substrates were cleaned in detergent, de-ionized water, acetone, and isopropanol sequentially by ultra-sonic bath for 30 minutes, respectively, and then dried in an even at 70 ℃ overnight. Further UV-Ozone treatment for 20 min was applied before use to improve its work function and clearance. Then the PEDOT:PSS (Heraeus Clevios P VP. AI 4083, filtered at 0.45 μm) was spin-coated onto the cleaned ITO-coated glass substrate at 5500 rpm for 30 s followed by annealing at 150 ℃ for 15 minutes in air to obtain ~30 nm thick film. The PEDOT:PSS-coated ITO substrates were then transferred into a N_2_-filled glove box for further device fabrication. The donor:acceptor blends solution with a weight ratio of 1:1.2 were dissolved in the CF, in which the solid concentration is 14.2 mg/mL in total, , with 1,3,5-Trichlorobenzene(TCB) as an additive. For ternary OSCs, PM6:L8-BO:BQx-MeCl with a weight ratio of 1:1:0.2 and 15.4 mg/ml total concentration also were dissolved in CF, using TCB as additives. Then the blend solution was stirred under 40 ℃ for 1 hours to mix intensively in a nitrogen-filled glove box. The blend solution was spin-casted at 2500 rpm for 30 s onto the PEDOT:PSS films followed by a thermal annealing of 100°C for 5 min. For all types of devices, a methanol with 0.5% vol acetic acid blend solution of PNDIT-F3N at a concentration of 0.5 mg mL^-1^ was spin-coated onto the active layer at 2000 rpm for 30s. The thickness was measured by a profilometer(KLA Tencor D-500)

The active layer thickness is ~110 nm measured by Bruker Dektak XT profilometer. A thin layer of PNDIT-F3N (~10 nm) was spin-coated onto the active layer, and Ag electrode (~100 nm) was deposited on top of the electron transfer layer in a thermal evaporator under vacuum of 1×10^-5^ Pa through a shadow mask. The current density-voltage (*J*-*V*) curves of all encapsulated devices were measured using a Keithley 2400 Source Meter under AM 1.5G (100 mW cm^-2^) using an Enlitech solar simulator. The light intensity was calibrated using a standard Si diode with KG5 filter to bring spectral mismatch to unity. Optical microscope (Olympus BX51) was used to define the device area (4.0 mm²). EQEs were measured using an Enlitech QE-S EQE system equipped with a standard Si diode. Monochromatic light was generated from a Enlitech 300W lamp source.

**Characterization Methods**

**Molecular structure optimization, orbital energy, and reorganization energy.** The neutral state molecular structure of BQx-MeCl, BQx-MeF, BQx-MeBr, BQx-Me2, and PM6 were optimized at level B3LYP-D3(BJ)/6-31G**, the anionic molecular structure of BQx-MeCl, BQx-MeF, BQx-MeBr, and BQx-Me_2_ were optimized at level B3LYP-D3(BJ)/6-31+G**, the cationic molecular structure of PM6 was optimized at level B3LYP-D3(BJ)/6-31G**, and all single-point energy were calculated at level ωB97xD/6-311+G**, using Gaussian 16, These ground-state structures exhibit no imaginary frequencies through vibrational frequency analysis. Revision A.03. Alkyl chains were substituted by methyl group. The molecular structure of PM6 was simplified into 2 repeating units. Internal reorganization energies of charge transfer were calculated using the four-point adiabatic formalism:

$$\lambda_{int}=\lambda_{N}+\lambda_{C}=\left( E_{N}^{q}-E_{N}^{0} \right)+\left( E_{C}^{0}-E_{C}^{q} \right)$$

where $E_{N}^{q}$ and $E_{N}^{0}$ indicate the energy for the neutral molecule and of its charged ion with the optimized geometry of the neutral molecule respectively; $E_{C}^{0}$and $E_{C}^{q}$ indicate the energy for the neutral molecule and of its charged ion with the optimized geometry of the charged ion respectively. The superscript q refer to the molecular charge. For BQx-MeCl, BQx-MeF, BQx-MeBr, and BQx-Me_2_, the molecular charge is -1; for PM6, the molecular charge is +1.

**Molecular Dynamic (MD) simulations**

The MD simulations were conducted utilizing the GROMACS (version 2018.4) software package.^[1,2]^ System temperature regulation was implemented through the velocity-rescale algorithm, while pressure equilibration was managed with the Berendsen barostat to maintain atmospheric pressure. The simulation framework incorporated three-dimensional periodic boundary conditions coupled with the General AMBER Force Field (GAFF)^[3]^ for molecular interactions. For electrostatic calculations, long-range electrostatic forces were evaluated through the Particle Mesh Ewald (PME) summation technique, with the cutoff distance for short-range interactions (electrostatic and van der Waals) set to 1.5 nm. Force field parameter refinement was achieved through vibrational frequency analysis using the Sobtop platform.^[4]^ Atomic charges were derived through the Restrained Electrostatic Potential (RESP) protocol, executed via the Multiwfn toolkit (v3.6)^[5]^.

The morphology of BQx-MeX (X=F, Cl, Br) films were simulated through a multi-stage protocol: (i) Firstly, cubic simulation boxes were created containing 300 non-fullerene acceptor (NFA) molecules randomly dispersed within 20,000 chloroform molecules, establishing a mixed NFA-solvent matrix; (ii) Than, the system underwent 40-ns NPT ensemble relaxation to achieve equilibrium molecular packing through spontaneous self-organization; (iii) A stepwise solvent removal protocol was implemented, where 100 chloroform molecules were radomly removed from the simulation box at intervals of 0.5 ns, mimicking experimental film-forming kinetics; (iv) Following complete solvent elimination, the residual acceptor was subjected to 10-ns NPT ensemble equilibration under ambient conditions to obtain stabilized acceptor films.

**UV–visible (UV-Vis) absorption**. UV-Vis absorption spectra of films were acquired on a Perkin Elmer Lambda 20 UV/VIS Spectrophotometer. The diluted solutions of BQx-MeF, BQx-MeCl and BQx-MeBr were kept at a low concentration of 10^-5^ M. All film samples were spin-cast on glass/ITO substrates.

**Thermogravimetric (TGA) analysis**. Thermogravimetric analysis (TGA) plots were measured with a Discovery series instrument under a nitrogen atmosphere at heating and cooling rates of 10 ℃ min^-1^.

**Cyclic voltammetry (CV)**. Cyclic voltammetry was carried out on a CHI610E electrochemical workstation with the three electrodes configuration, using Ag/AgCl as the reference electrode, a Pt plate as the counter electrode, and a glassy carbon as the working electrode. 0.1 mol L^-1^ tetrabutylammonium hexafluorophosphate in anhydrous acetonitrile was used as the supporting electrolyte. The polymer and small molecules were drop-cast onto the glassy carbon electrode from chloroform solutions (5 mg/mL) to form thin films. Potentials were referenced to the ferrocenium/ferrocene couple by using ferrocene as external standards in acetonitrile solutions. The scan rate is 100 mV s^-1^. The conversion of reduction/oxidation onsets and LUMO/HOMO energy levels can be described as: equation (2):*E*_LUMO_ = -[e(*E* ^red^–*E* ^Fc/Fc+^)+4.8]; equation (3):*E*_HOMO_ = -[e(*E* ^ox^–*E* ^Fc/Fc+^)+4.8].

**Atomic force microscopy (AFM)**. AFM measurements were performed by using a Scanning Probe MicroscopeDimension 3100 in tapping mode under atmosphere conditions at room temperature. All film samples were spin-cast on glass/ITO substrates.

**Grazing incidence wide angle X-ray scattering (GIWAXS)**. The Grazing-incidence wide-angle X-ray scattering (GIWAXS) measurement was carried out with a Xeuss 2.0 SAXS/WAXS laboratory beamline using a Cu X-ray source (8.05 keV, 1.54 Å) and a Pilatus3R 300 K detector. The incidence angle is 0.2°. The samples for GIWAXS measurement were fabricated on the silicon substrates using the same recipe for the devices.

**Photoluminescence (PL)**. The PL measurements were conducted by using FLS1000 equipment. The emission spectra of these three pure NFAs and BQx-MeF, BQx-MeCl and BQx-MeBr-based devices were obtained using the same setup used for recording electroluminescence spectra excited by the 514 nm and 785 nm wavelength provided by Xenon lamp. (Detector for NIR 5509 PMT, 600-1700nm).

**FTPS-EQE and EL-EQE measurements.** FTPS-EQE was measured using a integrated system (PECT-600, Enlitech), where the photocurrent was amplified and modulated by a lock-in instrument. EL-EQE measurements were performed by applying external voltage/current sources through the devices (REPS-Pro, Enlitech). All of the devices were prepared for EL-EQE measurements according to the optimal device fabrication conditions. EL-EQE measurements were carried out from 0 to 1.8 V.

∆*E*_1_=*E*_g_ – q*V*_OC_^SQ^

∆*E*_2_= q*V*_OC_^SQ^ - q*V*_OC_^rad^

∆*E*_3_=q*V*_OC_^rad^ – q*V*_OC_=-kTln(EQE_EL_)

**Transient photovoltage (TPV) and transient photocurrent (TPC) measurements.** In TPV measurements, the devices were placed under background light bias enabled by a focused Quartz Tungsten-Halogen Lamp with an intensity of similar to working devices, *i.e.,* the device voltage matches the open-circuit voltage under solar illumination conditions. Photo-excitations were generated with an 8 ns pulses from a laser system (Oriental Spectra, NLD520). The wavelength for the excitation was tuned to 518 nm with a spectral width of 3 nm. A digital oscilloscope was used to acquire the TPV signal at the open-circuit condition. TPC signals were measured under short-circuit conditions under the same excitation wavelength without background light bias. The TPV and TPC decay curves were fitted with a single exponential function, and the decay constants were obtained from the fitting.

**Charge Carrier Mobility Measurement**.

Hole-only diode configuration: glass/ITO/PEDOT:PSS/active layer/MoO_3_/Ag. Electron-only diode configuration: glass/ITO/ZnO/active layer/PNDIT-F3N/Ag. The mobility in active layers were determined by fitting the dark current hole/electron-only diodes to the space-charge limited current (SCLC) model. The mobility was determined by the equation:

𝐽 = 9𝜀_0_𝜀_𝑟_𝜇_0_𝑉^2^/ 8𝑑^3^

where *J* is current density, μ_0_ is the hole or electron mobility, ε_r_ is the dielectric permittivity of the active layer (generally assumed to be 3 for organic materials), ε0 is the dielectric permittivity of free space (ε_0_ = 8.854×10^-12^ F m^-1^), d is the film thickness of active layers, and *V* is the voltage, which is defined as *V* = *V*_appl_ - *V*_bi_, where *V*_appl_ is the applied voltage, *V*_bi_ is the built-in voltage.

**Transient absorption spectroscopy.** Measurements were performed using a homebuilt experimental setup with an amplified Ti:sapphire laser (Coherent Legend Elite), with pulse duration of 120 fs, centered at 800 nm and at a repetition rate of 1000 Hz. The pump pulses were generated using an optical parametric amplifier (Coherent Opera Solo) and then chopped 500 Hz. The probe beam was traversed a mechanical translation stage, enabling a time delay of up to 2 ns between pump and probe pulses, and then was focused on an Yttrium Aluminium Garnet crystal plate. After passing through the photoexcited sample, the probe pulses were spectrally dispersed using grating and then collected with a silicon line CCD (Hamamatsu S8380) (visible components, 500-1000 nm range) or an InGaAs line CCD (Hamamatsu G11620) (IR components, 800-1600 nm range). The differential transmission signals at various delay times were calculated from the sequential probe shots corresponding to the pump on and off cases as ((T_pump_ _ON_ – T_pump_ _OFF_)/T_pump_ _OFF_). Measurements were conducted under excitation in fundamental absorption band of the acceptor (800 nm) with an average flux of ~2µJ cm^-2^. The decomposition of TA spectra was carried out via soft-modelling by multivariate curve resolution-alternating least square (MCR-ALS) method with a singular value decomposition parameters as an initial guess.

**In-situ UV-Vis absorption measurements**. In-situ UV-Vis absorption measurements were carried out with a DU-100 system. The optical fiber was aligned so that the center of the light is focused on the center of the film. The sampling interval was 5 ms with an integration time of 5 ms per sample point. The average sampling times were adjusted between 7 times to obtain smooth absorption curves, then, the actual sampling interval is 70ms.

**Multichannel maximum power point tracking (MPPT) measurements.** The devices photostabilities were measured by using the solar cell stability test system (PR-SCCS, PURI Materials, China), and PURI2400-8Q as the source meters to record the solar-cells characteristics in an N_2_ atmosphere with 45℃, which were conducted under continuous 100 mw/cm2 illumination provided by LED-solar simulators (PR-LEDSUN-8C, PURI Materials, China, with spectrum ranging from 350nm to 900nm). And the results were automatically recorded by the aging test software (PR-SCCS-MPPT, PURI Materials, China).

**2. Synthesis of BQx-MeF, BQx-MeCl and BQx-MeB**r**.**

For the synthetic route of BQx-MeF, BQx-MeCl and BQx-MeBr were synthesized according to the literature method.^[6,7]^

**Scheme S1.** The overall synthetic route to BQx-MeF, BQx-MeCl and BQx-MeBr.

**Synthesis of compound 2a**, **2b** and **2c**.

To a solution of **1** (500 mg, 0.46 mmol, 1.0 eq.) in 50 ml of tetrahydrofuran was added LiAlH_4_ (175 mg, 4.60 mmol, 10 eq.) under an atmosphere of nitrogen. The reaction mixture was kept stirring at 0 °C for 30 minutes. The reaction mixture was heated to 82 °C for 12 hours and then quenched with water. The resulting mixture was extracted with CH_2_Cl_2_ and the organic layer was washed with brine, dried with anhydrous MgSO_4_ and concentrated under a reduced pressure. The crude mixture was used for next step without further purification. To a solution of crude mixture in 40 ml of chloroform was added sequentially 2,3-dichloro-5,6-dicyano-1,4-benzoquinone (DDQ, 118.76 mg, 0.53 mmol, 1.15 eq.) and 4-fluoro-5-methylbenzene-1,2-diamine (322 mg, 2.30 mmol, 5.0 eq.). The reaction mixture was kept stirred at room temperature under the atmosphere of nitrogen for 6 hours. The resulting mixture was concentrated under a reduced pressure and purified by column chromatography on silica gel with hexane/dichloromethane as eluent to afford **2a** as a purplish red solid (363 mg, 68%). **2b** and **2c** were synthesized by a similar method with a yield of 68% and 67% respectively.

**2a**: ^1^H NMR (400 MHz, CDCl_3_, *δ*/ppm): 8.3 (d, *J* = 12.0 Hz, 1H), 8.04 (d, *J* = 8.0 Hz, 1H), 7.03 (s, 2H), 4.70 (d, *J* = 8.0 Hz, 4H), 2.89 (t, *J* = 16.0 Hz, 4H), 2.65 (s, 2H), 2.22-2.19 (m, 2H), 1.96-1.89 (m, 4H), 1.66-1.31 (m, 34H), 1.19-0.80 (m, 56H), 0.72-0.68 (m, 6H). ^13^C NMR (100 MHz, CDCl_3_, *δ*/ppm): 162.24, 159.74, 142.40, 142.38, 140.40, 140.27, 138.16, 137.55, 137.16, 137.14, 136.34, 136.29, 136.26, 131.00, 130.66, 129.96, 129.90, 128.75, 128.53, 122.90, 122.75, 122.67, 118.23, 118.20, 116.57, 116.30, 110.93, 110.71, 54.43, 38.11, 31.37, 31.22, 31.05, 29.90, 29.81, 29.16, 29.13, 29.11, 29.09, 29.00, 28.94, 28.85, 28.82, 28.79, 28.65, 28.58, 28.36, 24.94, 22.13, 22.09, 22.01, 21.89, 15.16, 15.13, 13.55, 13.50, 13.37. HRMS (ESI, m/z): [M^+^], calcd. for C_79_H_119_FN_4_S_4_: 1272.0900; found:1272.8412.

**2b**: ^1^H NMR (400 MHz, CDCl_3_, *δ*/ppm): 8.48 (s, 1H), 8.37 (s, 1H), 7.03 (s, 2H), 4.69 (d, *J* = 8.0 Hz, 4H), 2.88 (t, *J* = 16.0 Hz, 4H), 2.72 (s, 2H), 2.21-2.18 (m, 2H), 1.96-1.86 (m, 4H), 1.53-1.31 (m, 34H), 1.18-0.79 (m, 56H), 0.71-0.67 (m, 6H). ^13^C NMR (100 MHz, CDCl_3_, *δ*/ppm):142.45, 142.39, 139.70, 139.49, 137.79, 137.68, 136.30, 136.26, 136.17, 134.85, 130.95, 130.91, 129.11, 127.51, 122.90, 122.86, 122.79, 122.75, 118.26, 116.43, 116.40, 67.36, 54.44, 38.11, 31.36, 31.21, 31.03, 19.89, 29.81, 29.28, 29.16, 29.12, 29.10, 29.00, 28.93, 28.84, 28.81, 28.78, 28.57, 28.48, 28.35, 25.02, 24.94, 22.13, 22.01, 21.95, 21.88, 20.19, 13.55, 13.50, 13.37, 13.31. HRMS (ESI, m/z): [M^+^], calcd. for C_79_H_119_ClN_4_S_4_: 1288.5400; found:1287.8082.

**2c**: ^1^H NMR (400 MHz, CDCl_3_, *δ*/ppm): 8.70 (s, 1H), 8.31 (s, 1H), 7.03 (s, 2H), 4.72 (d, *J* = 8.0 Hz, 4H), 2.91-2.86 (m, 4H), 2.74 (s, 2H), 2.26-2.22 (m, 2H), 1.96-1.89 (m, 4H), 1.54-1.32 (m, 34H), 1.19-0.80 (m, 56H), 0.72-0.63 (m, 6H). ^13^C NMR (100 MHz, CDCl_3_, *δ*/ppm):142.49, 142.43, 139.83, 137.90, 137.65, 137.34, 136.31, 136.30, 136.25, 131.20, 130.99, 130.89, 128.77, 125.32, 122.90, 122.85, 122.83, 122.80, 118.26, 118.23, 116.46, 54.47, 38.14, 31.39, 31.24, 31.07, 29.93, 29.85, 29.19, 29.16, 29.13, 29.04, 28.97, 28.88, 28.84, 28.82, 28.61, 28.37, 24.98, 22.91, 22.16, 22.04, 21.91, 13.58, 13.53, 13.40. HRMS (ESI, m/z): [M^+^], calcd. for C_79_H_119_BrN_4_S_4_: 1332.9900; found:1333.7554.

**Synthesis of compound 3a, 3b and 3c.**

Phosphorus oxychloride (2 mL) was added at 0 °C to anhydrous DMF (10 mL) and the resulting mixture was stirred for 1 h. To a solution of **2a** (0.20 g, 0.17 mmol) in 15 ml of 1,2-dichloroethane was added the mixture dropwise. After stirring for additional 30 minutes at 0 °C, the mixture was then heated to 85 °C for overnight and then quenched by 20 ml of 1 M NaOH solution. The reaction mixture was cooled and partitioned between dichloromethane and water. The aqueous layer was extracted with dichloromethane twice. The combined organic layer was washed with brine, dried with anhydrous MgSO_4_, and concentrated under reduced pressure. The crude mixture was purified by column chromatography on silica gel with hexane/dichloromethane as eluent to afford **3a** as an orange solid. (182 mg, 88%). **3b** and **3c** were synthesized by a similar method with a yield of 86% and 85% respectively.

**3a:** ^1^H NMR (400 MHz, CDCl_3_, *δ*/ppm): 10.15 (s, 2H), 8.21 (d, *J* = 8.0 Hz, 1H), 7.94 (d, *J* = 8.0 Hz, 1H), 4.70 (d, *J* = 4.0 Hz, 4H), 3.22 (t, *J* = 16.0 Hz, 4H), 2.62 (s, 2H), 2.15 (s, 2H), 1.99-1.91 (m, 4H), 1.54-1.26 (m, 34H), 1.14-0.75 (m, 56H), 0.68-0.64 (m, 6H). ^13^C NMR (100 MHz, CDCl_3_, *δ*/ppm):181.06, 162.59, 160.07, 146.32, 146.27, 143.48, 140.69, 140.55, 138.40, 137.35, 136.93, 136.91, 136.08, 136.06, 136.00, 132.11, 131.84, 129.88, 129.82, 129.78, 129.56, 128.98, 128.93, 127.45, 127.33, 117.42, 117.18, 110.90, 110.68, 54.64, 38.55, 38.33, 31.29, 31.13, 30.94, 29.92, 29.85, 29.76, 29.10, 29.08, 29.06, 29.01, 28.95, 28.90, 28.82, 28.80, 28.73, 28.68, 28.52, 27.56, 24.90, 22.06, 21.94, 21.84, 15.18, 15.14, 13.49, 13.44, 13.32. HRMS (ESI, m/z): [M^+^], calcd. for C_81_H_119_FN_4_O_2_S_4_: 1328.1100; found:1327.8241.

**3b:** ^1^H NMR (400 MHz, CDCl_3_, *δ*/ppm): 10.15 (s, 2H), 8.35 (s, 1H), 8.21 (s, 1H), 4.69 (d, *J* = 8.0 Hz, 4H), 3.24-3.18 (m, 4H), 2.68 (s, 2H), 2.17-2.14 (s, 2H), 1.99-1.89 (m, 4H), 1.54-1.27 (m, 34H), 1.13-0.74 (m, 56H), 0.68-0.64 (m, 6H). ^13^C NMR (100 MHz, CDCl_3_, *δ*/ppm):181.11, 146.35, 146.31, 143.51, 143.49, 139.87, 139.69, 137.57, 137.47, 137.15, 136.08, 136.05, 136.01, 136.00, 135.70, 132.08, 132.01, 128.98, 128.95, 128.89, 127.48, 127.42, 127.36, 117.27, 54.71, 38.39, 38.35, 31.29, 31.13, 30.94, 30.81, 29.94, 29.91, 29.87, 29.81, 29.76, 29.56, 29.11, 29.09, 29.07, 29.02, 28.96, 28.83, 28.81, 28.73, 28.70, 28.53, 27.57, 24.92, 24.88, 22.07, 21.94, 21.84, 20.22, 13.50, 13.44, 13.33. HRMS (ESI, m/z): [M^+^], calcd. for C_81_H_119_ClN_4_O_2_S_4_: 1344.5600; found:1344.7960.

**3c:** ^1^H NMR (400 MHz, CDCl_3_, *δ*/ppm): 10.14 (d, *J* = 8.0 Hz, 2H), 8.26 (s, 1H), 7.97 (s, 1H), 4.74 (s, 4H), 3.19-3.06 (m, 4H), 2.52 (s, 2H), 2.24 (s, 2H), 1.96-1.79 (m, 4H), 1.52-0.83 (m, 84H), 0.76-0.58 (m, 12H). ^13^C NMR (100 MHz, CDCl_3_, *δ*/ppm): 180.98, 146.18, 146.13, 143.55, 143.51, 139.69, 139.58, 137.97, 137.30, 137.10, 136.02, 135.91, 135.89, 135.83, 131.62, 130.60, 128.88, 128.68, 128.28, 127.43, 127.30, 125.85, 117.26, 117.18, 38.59, 38.52, 31.30, 31.15, 31.12, 31.04, 31.01, 30.04, 29.99, 29.90, 29.24, 29.19, 29.16, 29.09, 29.07, 29.03, 29.00, 28.95, 28.86, 28.83, 28.80, 28.76, 28.74, 28.57, 28.54, 27.54, 27.48, 25.17, 25.12, 22.78, 22.08, 21.95, 21.93, 21.88, 13.51, 13.43, 13.41, 13.35, 13.33. HRMS (ESI, m/z): [M^+^], calcd. for C_81_H_119_BrN_4_O_2_S_4_: 1389.0100; found:1388.7405.

**Synthesis of BQx-MeF, BQx-MeCl and BQx-MeBr.**

To a mixture of **3a** (180 mg, 0.15 mmol), 5,6-difluoro-3-(dicyanomethylidene)-1-one (136 mg, 0.59 mmol), and pyridine (1 mL) was added 10 mL of chloroform. The resulting mixture was refluxed for 2 hours. The resulting mixture was concentrated and directly purified by column chromatography on silica gel with hexane/dichloromethane as an eluent to afford **BQx-MeF** as a black solid (168 mg, 64%). **BQx-MeCl** and **BQx-MeBr** were synthesized by a similar method with a yield of 67% and 65% respectively.

**BQx-MeF:** ^1^H NMR (400 MHz, CDCl_3_, *δ*/ppm): 8.88 (d, *J* = 8.0 Hz, 2H), 8.23-8.16 (m, 2H), 7.90 (d, *J* = 8.0 Hz, 1H), 7.70-7.61 (m, 3H), 4.93-4.79 (m, 4H), 3.15-3.14 (m, 4H), 2.56 (s, 2H), 2.45 (s, 2H), 1.81 (s, 4H), 1.51-1.10 (m, 78H), 0.87-0.83 (m, 6H), 0.78-0.66 (m, 12H). ^13^C NMR (100 MHz, CDCl_3_, *δ*/ppm):185.42, 160.25, 157.43, 153.31, 153.28, 152.34, 152.29, 152.20, 152.16, 145.81, 140.66, 140.53, 138.30, 137.15, 137.12, 136.71, 136.70, 135.83, 135.76, 135.56, 135.51, 133.96, 133.75, 133.70, 132.94, 132.70, 132.49, 130.76, 130.62, 130.56, 118.91, 118.66, 118.52, 114.30, 114.13, 113.71, 67.06, 55.02, 39.01, 31.30, 31.21, 31.14, 31.12, 31.01, 30.82, 30.06, 29.48, 29.35, 29.32, 29.14, 29.09, 29.05, 29,03, 29.00, 28.95, 28.93, 28.85, 28.76, 28.74, 28.69, 28.63, 28.59, 25.33, 25.28, 22.06, 22.02, 21.97, 21.88, 15.16, 15.13, 13.48, 13.46, 13.45, 13.42, 13.40. HRMS (ESI, m/z): [M^+^], calcd. for C_105_H_123_F_5_N_8_O_2_S_4_: 1752.4300; found:1752.8665.

**BQx-MeCl:** ^1^H NMR (400 MHz, CDCl_3_, *δ*/ppm): 8.95 (d, *J* = 12.0 Hz, 2H), 8.34-8.30 (m, 2H), 8.03 (s, 1H), 7.91 (s, 1H), 7.72-7.68 (m, 2H), 4.90 (d, *J* = 4.0 Hz, 4H), 3.18-3.10 (m, 4H), 2.60 (s, 2H), 2.39 (s, 2H), 1.87-1.79 (m, 4H), 1.60-1.00 (m, 78H), 0.87-0.82 (m, 6H), 0.76-0.67 (m, 12H). ^13^C NMR (100 MHz, CDCl_3_, *δ*/ppm):185.61, 161.15, 158.12, 137.06, 136.46, 136.01, 135.50, 134.57, 133.86, 133.24, 132.56, 131.10, 119.12, 118.89, 114.40, 114.00, 111.81, 107.13, 67.63, 38.69, 31.28, 31.00, 29.99, 29.08, 28.93, 28.58, 20.26, 13.42, 13.38. HRMS (ESI, m/z): [M^+^], calcd. for C_105_H_123_ClF_4_N_8_O_2_S_4_: 1768.8800; found:1768.8286.

**BQx-MeBr:** ^1^H NMR (400 MHz, CDCl_3_, *δ*/ppm): 8.86 (d, *J* = 20.0 Hz, 2H), 8.24-8.19 (m, 2H), 8.13 (s, 1H), 7.79 (s, 1H), 7.73-7.68 (m, 2H), 4.96-4.90 (m, 4H), 3.12-3.06 (m, 4H), 2.57 (s, 2H), 2.47 (s, 2H), 1.81-1.78 (m, 4H), 1.49-1.00 (m, 78H), 0.88-0.82 (m, 6H), 0.77-0.67 (m, 12H). ^13^C NMR (100 MHz, CDCl_3_, *δ*/ppm):185.42, 157.40, 154.78, 153.17, 152.39, 152.20, 145.90, 139.64, 138.91, 137.31, 137.09, 135.80, 135.52, 133.96, 133.72, 132.87, 132.84, 132.50, 130.84, 130.74, 128.19, 126.82, 118.68, 118.56, 114.30, 114.21, 113.67, 111.82, 111.61, 67.12, 67.05, 55.35, 39.17, 31.30, 31.21, 31.16, 30.82, 30.20, 29.55, 29.41, 29.38, 29.21, 29.14, 29.05, 28.99, 28.96, 28.84, 28.76, 28.69, 25.51, 25.44, 22.85, 22.06, 22.01, 21.97, 13.47, 13.45, 13.42, 13.39. HRMS (ESI, m/z): [M^+^], calcd. for C_105_H_123_BrF_4_N_8_O_2_S_4_: 1813.3300; found:1813.2633.

**3. Supplementary Figu6res (S1-S40).**

**3.1 Photophysical properties of BQx-MeF, BQx-MeCl and BQx-MeBr.**

**Figure. S1.** (a) Thermogravimetric analysis (TGA) curves of BQx-MeF, BQx-MeCl, and BQx-MeBr. (b) Normalized UV-Vis absorption spectra of the three electron acceptors in chloroform solution, in comparison with their photoluminescence (PL) spectra (with the Stokes shift denoted). (c) Normalized UV-Vis absorption spectra of the three SMAs in blend films. PL spectra excited at (d) 785 nm of BQx-MeF, BQx-MeCl and BQx-MeBr-based blend films and (e) 514 nm and 785 nm of BQx-MeF, BQx-MeCl and BQx-MeBr-based blend films. (f) CV curves of BQx-MeF, BQx-MeCl, and BQx-MeBr films.

**Figure. S2.** Theoretical density distribution for the frontier molecular orbits of BQx-MeF, BQx-MeCl and BQx-MeBr calculated at the B3LYP-D3(BJ)/6-31G** level.

**Figure. S3.** (a) *J*-*V* curves for different speeds based on the PM6:BQx-MeF, PM6:BQx-MeCl and PM6:BQx-MeBr. (b-e) Normal distributions of PCEs, *V*_OC_s, *J*_SC_s and FFs for 15 independent devices based on the PM6:BQx-MeF, PM6:BQx-MeCl and PM6:BQx-MeBr.

**Figure. S4.** Semilogarithmic plots of normalized EL, measured EQE, and EQE calculated by FTPS (EQE_FTPS_) as a function of energy for devices based on (a), PM6:BQx-MeF, (b), PM6:BQx-MeCl and (c), PM6:BQx-MeBr.

**Figure. S5**. (a) hole-only (b) electron-only device based on BQx-MeF, BQx-MeCl and BQx-MeBr.

**Figure S6.** (a) *J*_SC_ versus light intensity. (b) photo-CELIV plots of the devices. (c) IS curves of the PM6:SMAs based OSCs. (d) TPC decay curves. (e) TPV decay curves. (f) Normalized PCEs expressed as a function of light-soaking time under MPP tracking

**Figure. S7.** The in-situ UV-Vis absorption line profiles and of the (a) BQx-MeF-, (b) BQx-MeCl- and (c) BQx-MeBr-based blends. Time-resolved UV-Vis absorption spectra of (d) BQx-MeF-, (e) BQx-MeCl- and (f) BQx-MeBr-based blends.

**Figure. S8.** (a-c) 2D GIWAXS patterns and (d) 1D line-cuts of the the BQx-MeF, BQx-MeCl, BQx-MeBr and AFM (e-g) BQx-MeF, BQx-MeCl and BQx-MeBr-based device phase images; (h-m) height images of BQx-MeF, BQx-MeCl and BQx-MeBr in CF and toluene solution; (n-s) phase images of BQx-MeF, BQx-MeCl and BQx-MeBr in CF and toluene solution.


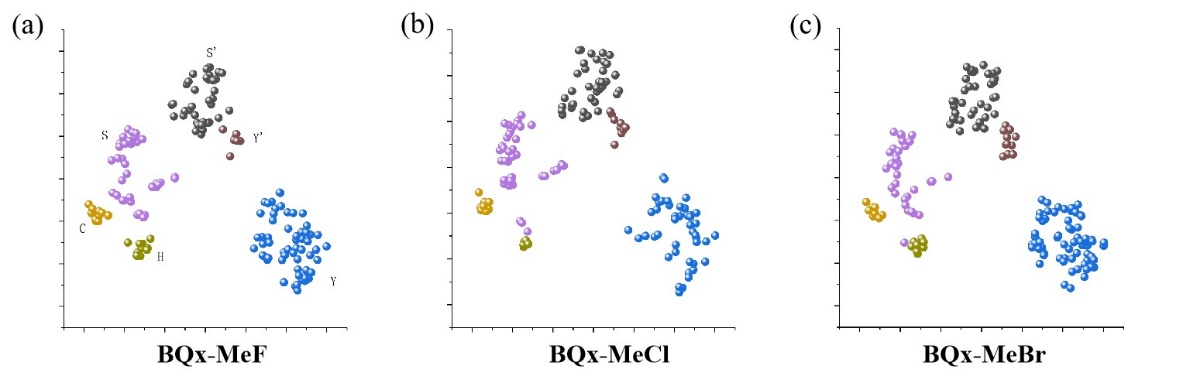


**Figure. S9. T**he classification of stacking structures for (a) BQx-MeF, (b) BQx-MeCl, (c) BQx-MeBr pristine films, respectively. The stacking structures are classified by agglomerative clustering method and reduce dimensionality by t-SNE.

**Figure. S10.** Pure acceptor TA spectra at 800 nm excitation: (a) BQx-MeF, (b) BQx-MeCl, (c) BQx-MeBr

**Figure. S11.** Comparison of raw TA data (a-c) with the spectral component corresponded to local exciton from decomposed TA spectra (d-f) of pure acceptor ((a,d) BQx-MeCl, (b,e) BQx-MeF, (c,f) BQx-MeBr ) and corresponding blends.

**3.2 Photovoltaic device performance.**

**Figure. S12**. Structure of compound PM6.

**3.3 NMR spectra and HR-MS**


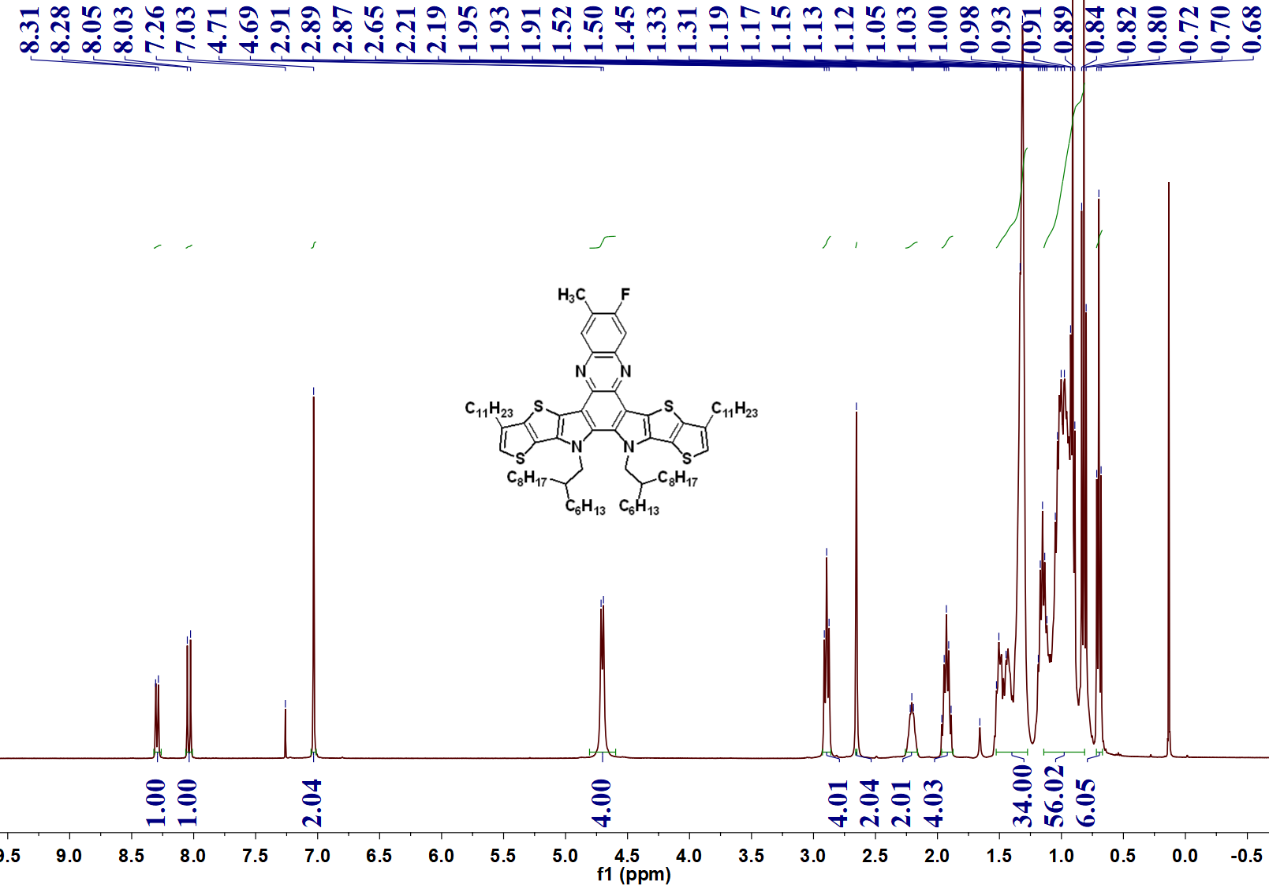


**Figure. S13. ^1^H NMR spectrum of compound 2a.**


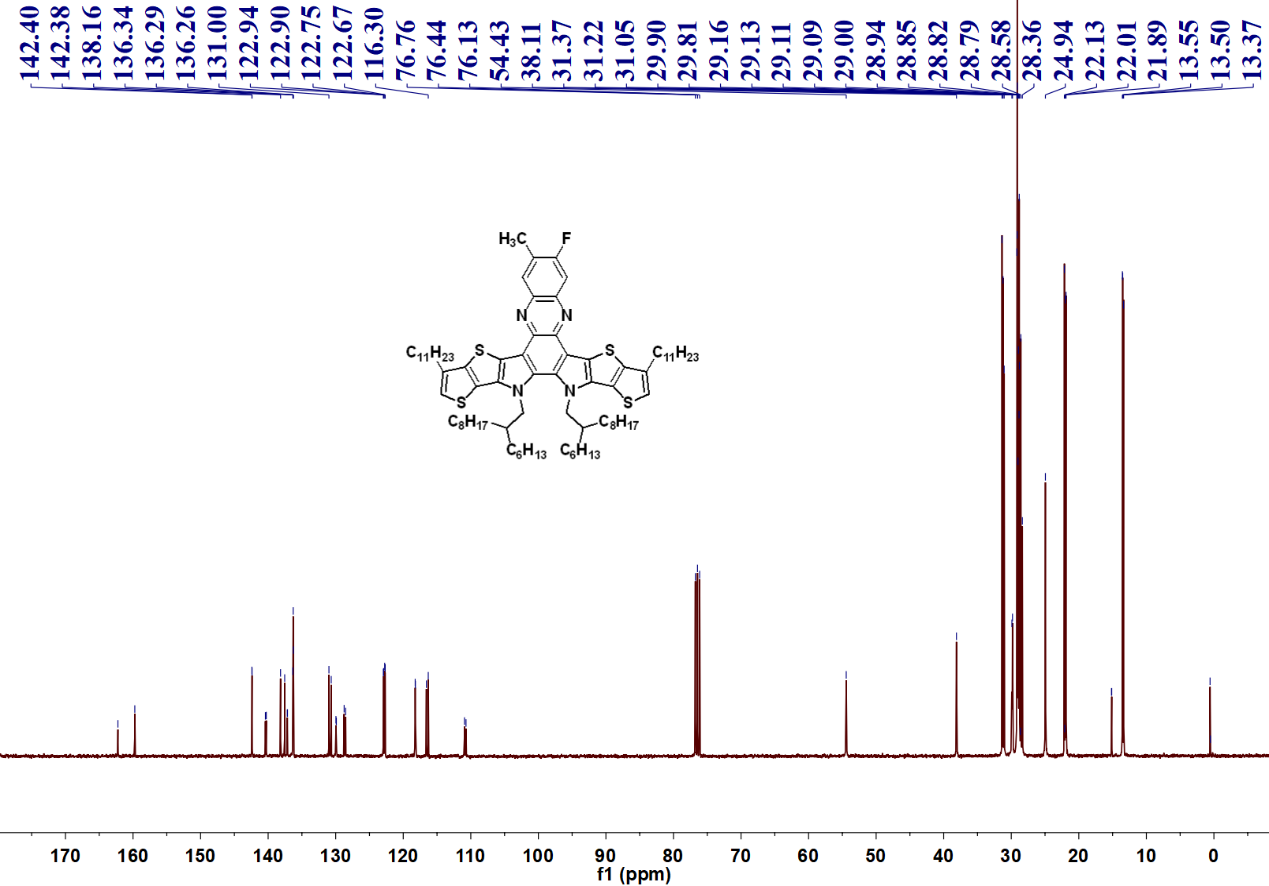


**Figure. S14. ^13^C NMR spectrum of compound 2a.**

**
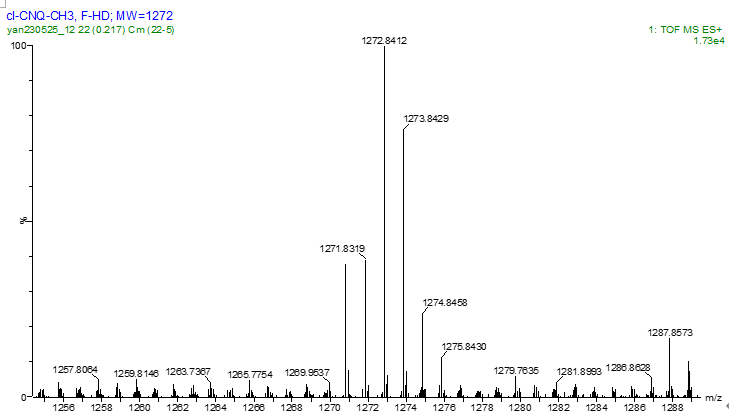
**

**Figure. S15. HRMS of compound 2a.**


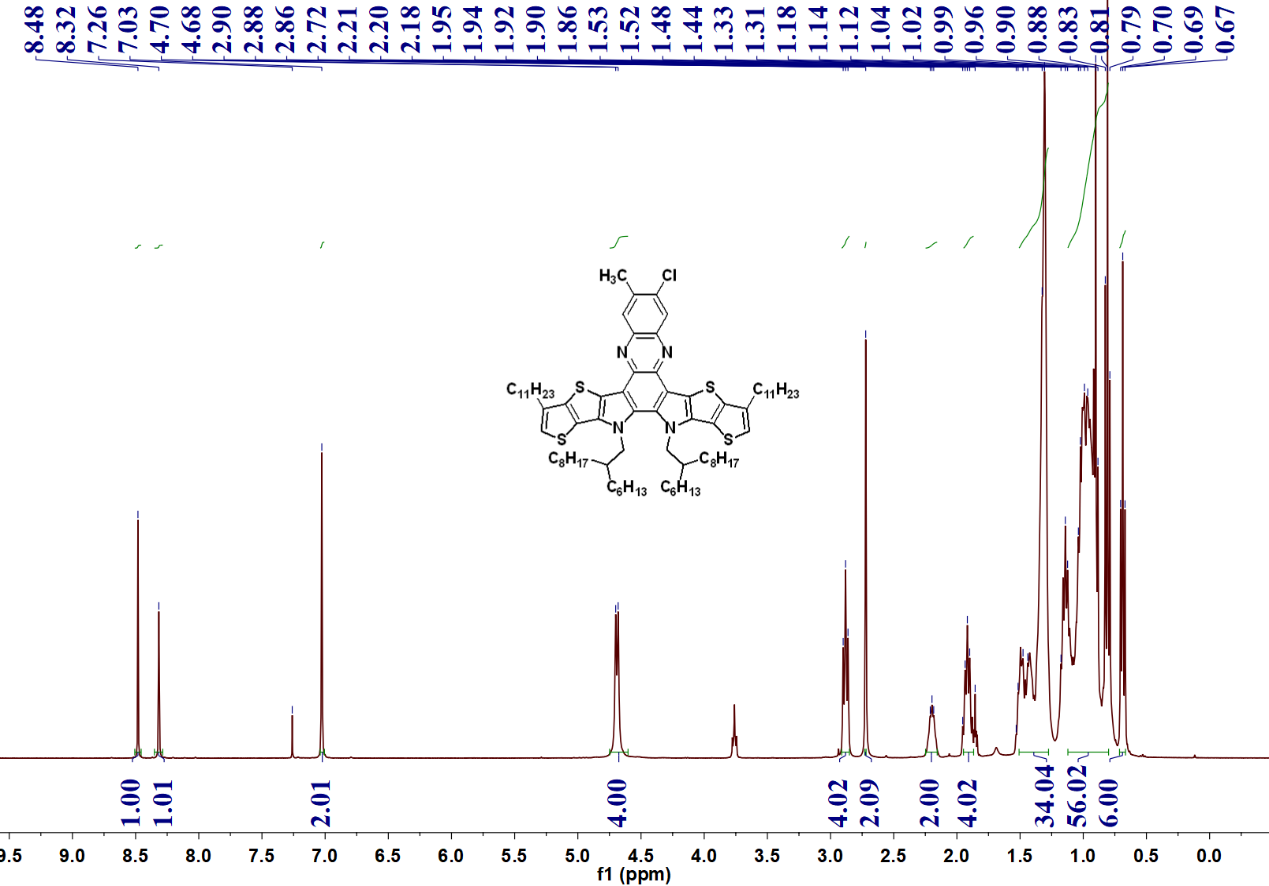


**Figure. S16. ^1^H NMR of compound 2b.**


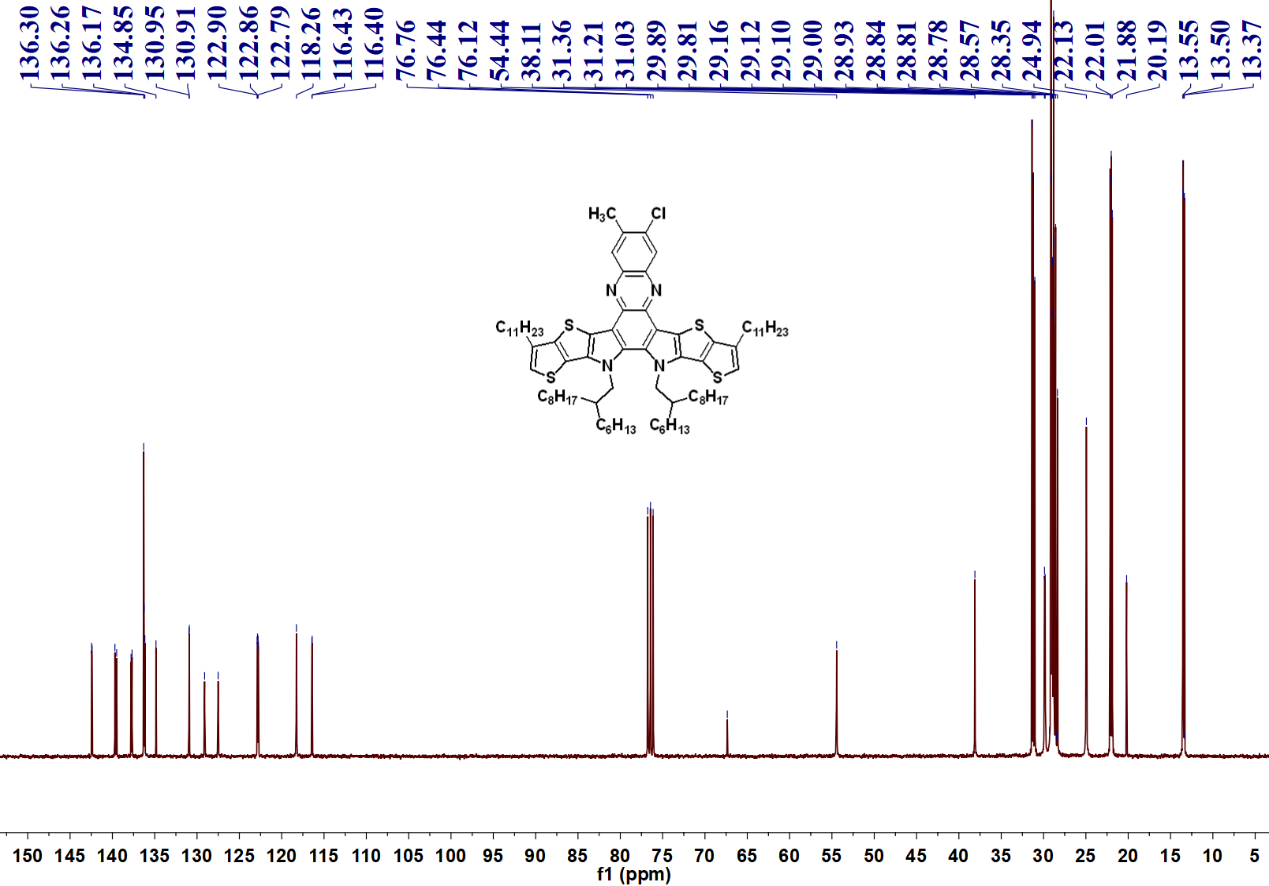


**Figure. S17. ^13^C NMR spectrum of compound 2b.**

**
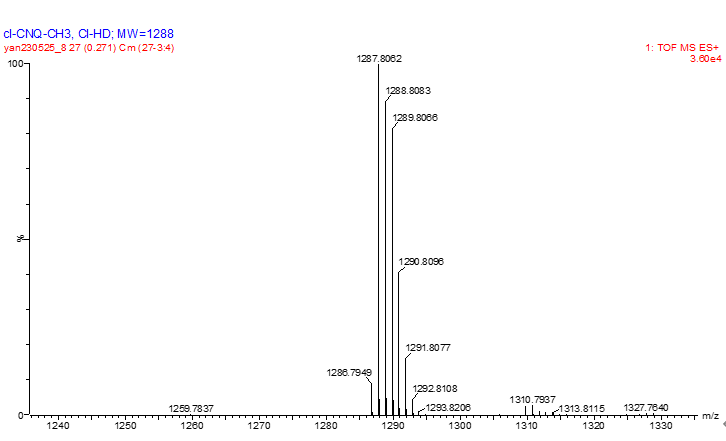
**

**Figure. S18. HRMS of compound 2b.**


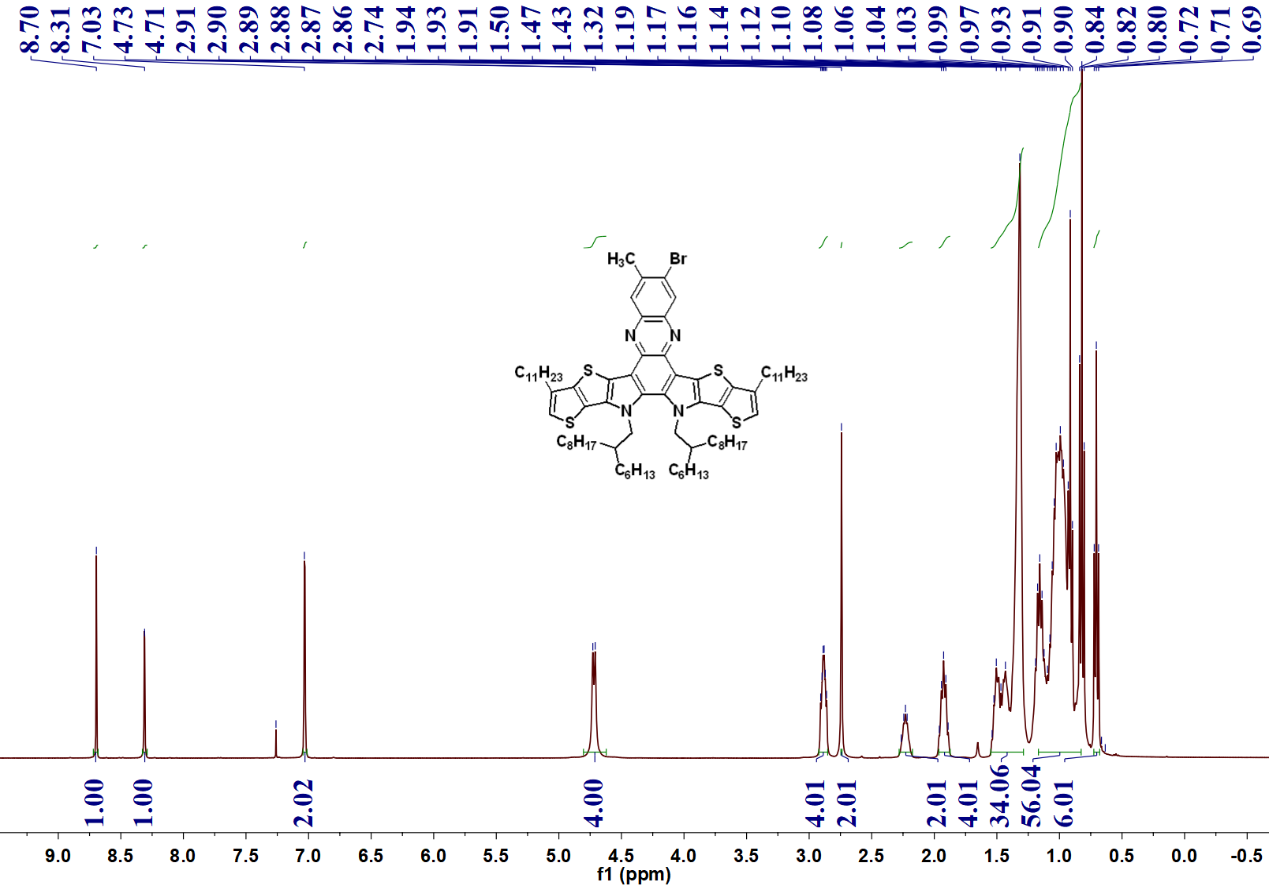


**Figure. S19. ^1^H NMR spectrum of compound 2c.**


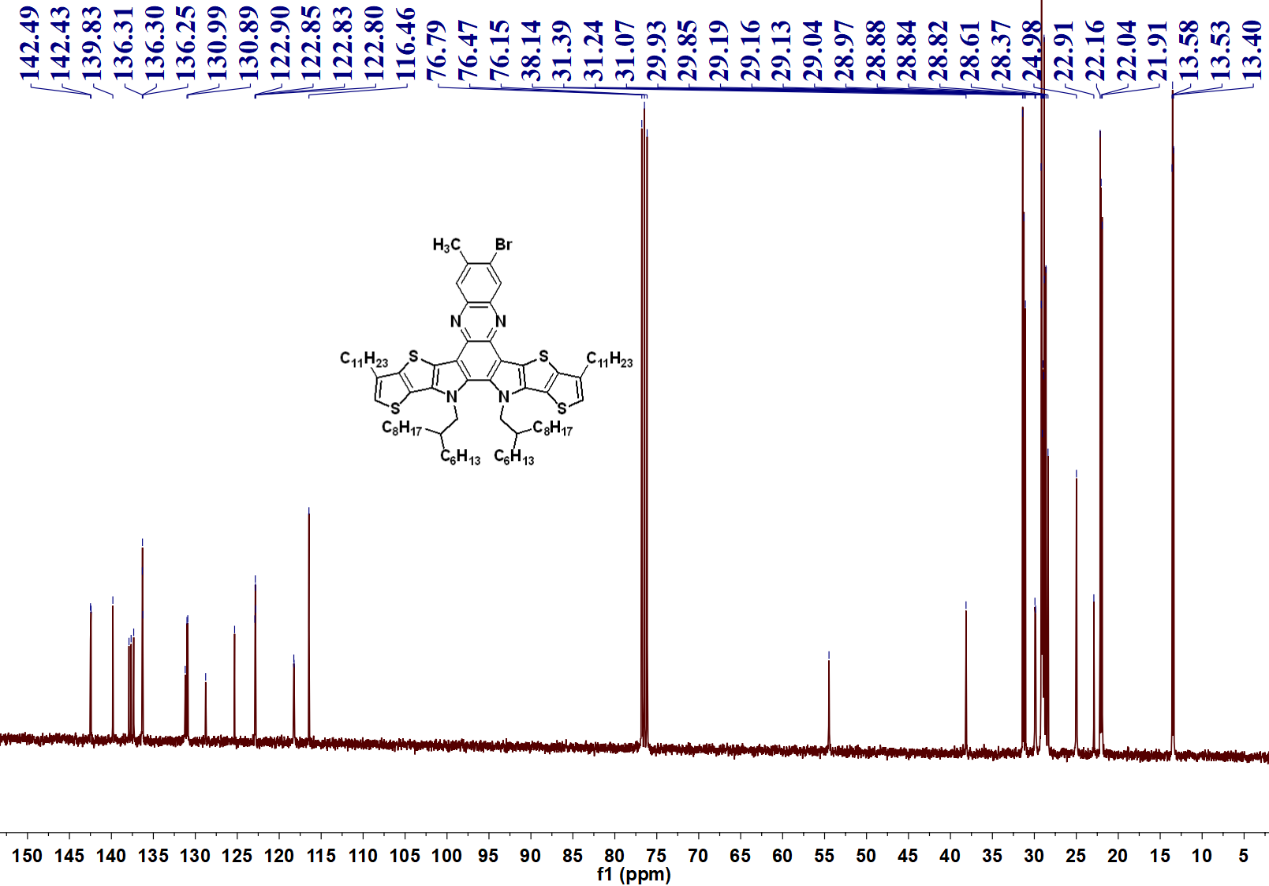


**Figure. S20. ^13^C NMR spectrum of compound 2c.**

**
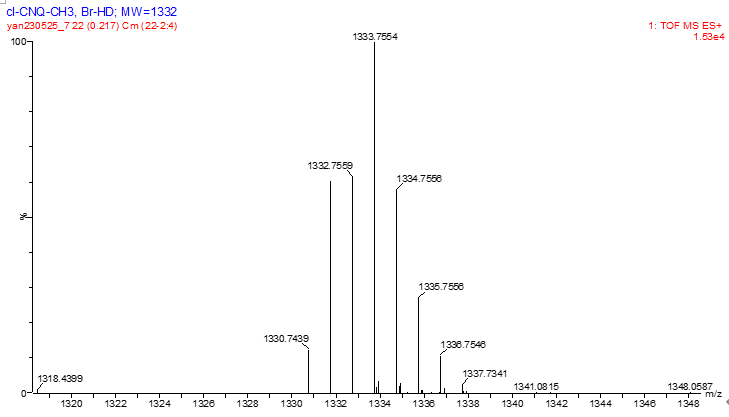
**

**Figure. S21. HRMS of compound 2c.**


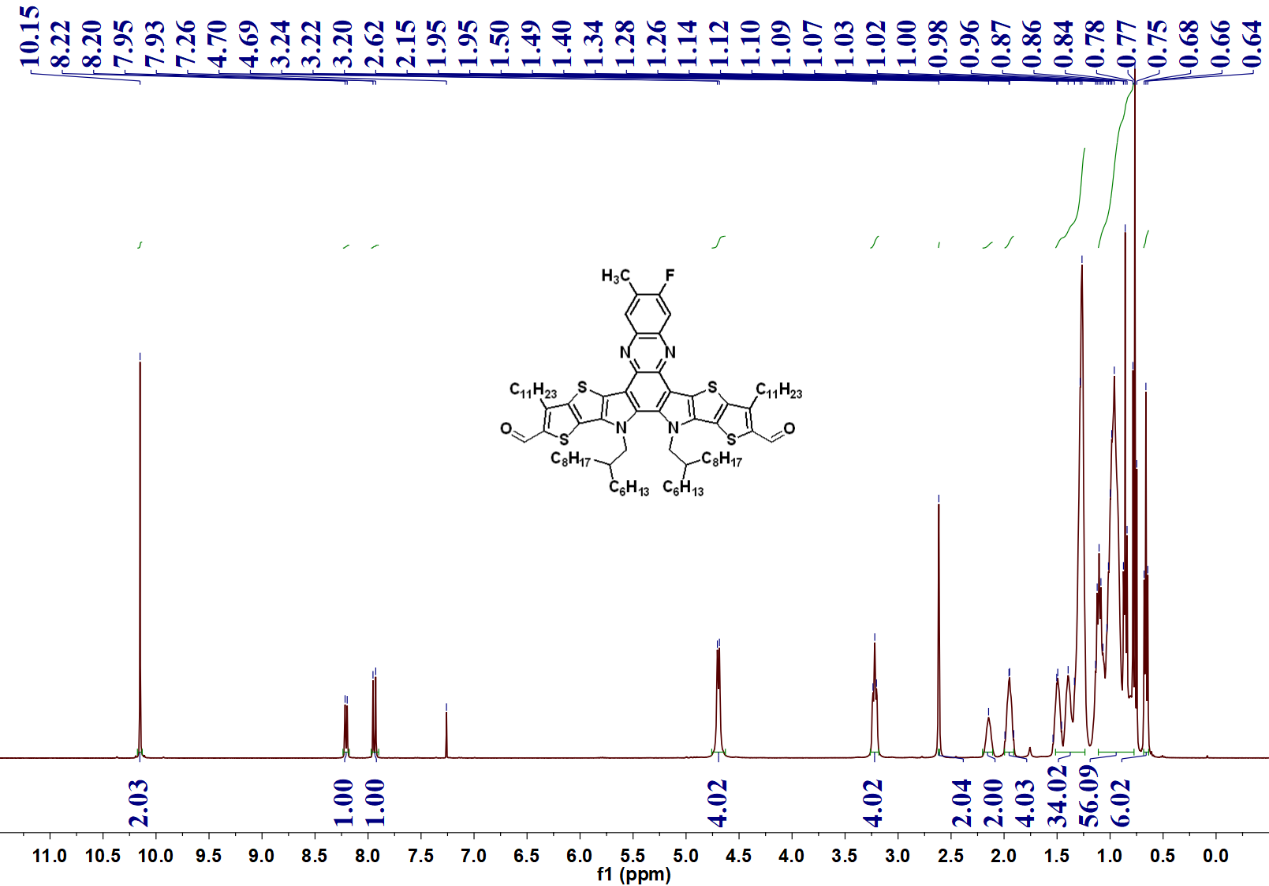


**Figure. S22. ^1^H NMR spectrum of compound 3a.**


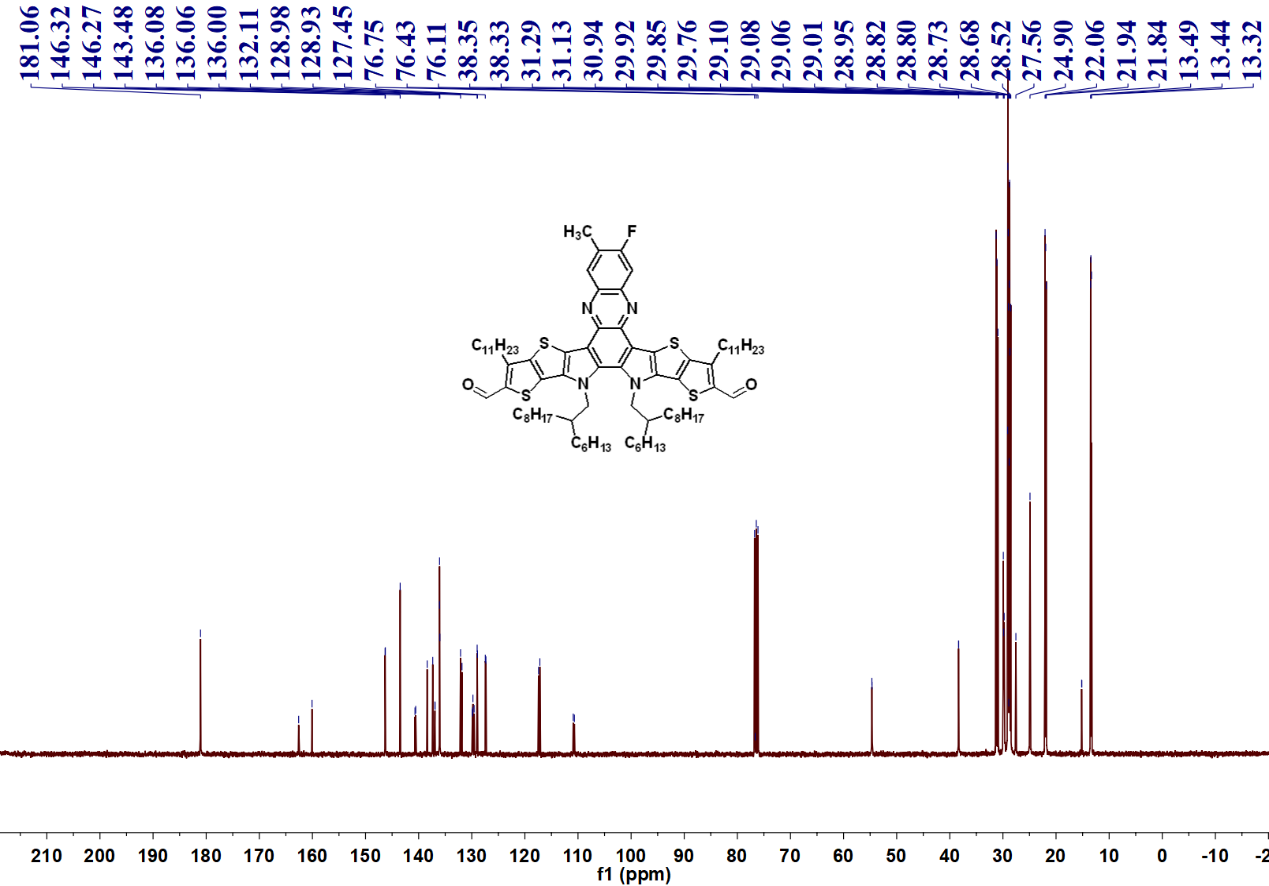


**Figure. S23. ^13^C NMR spectrum of compound 3a.**


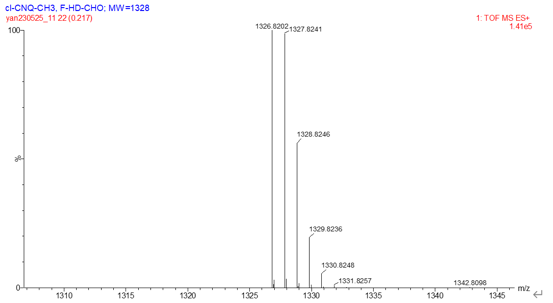


**Figure. S24. HRMS of compound 3a.**


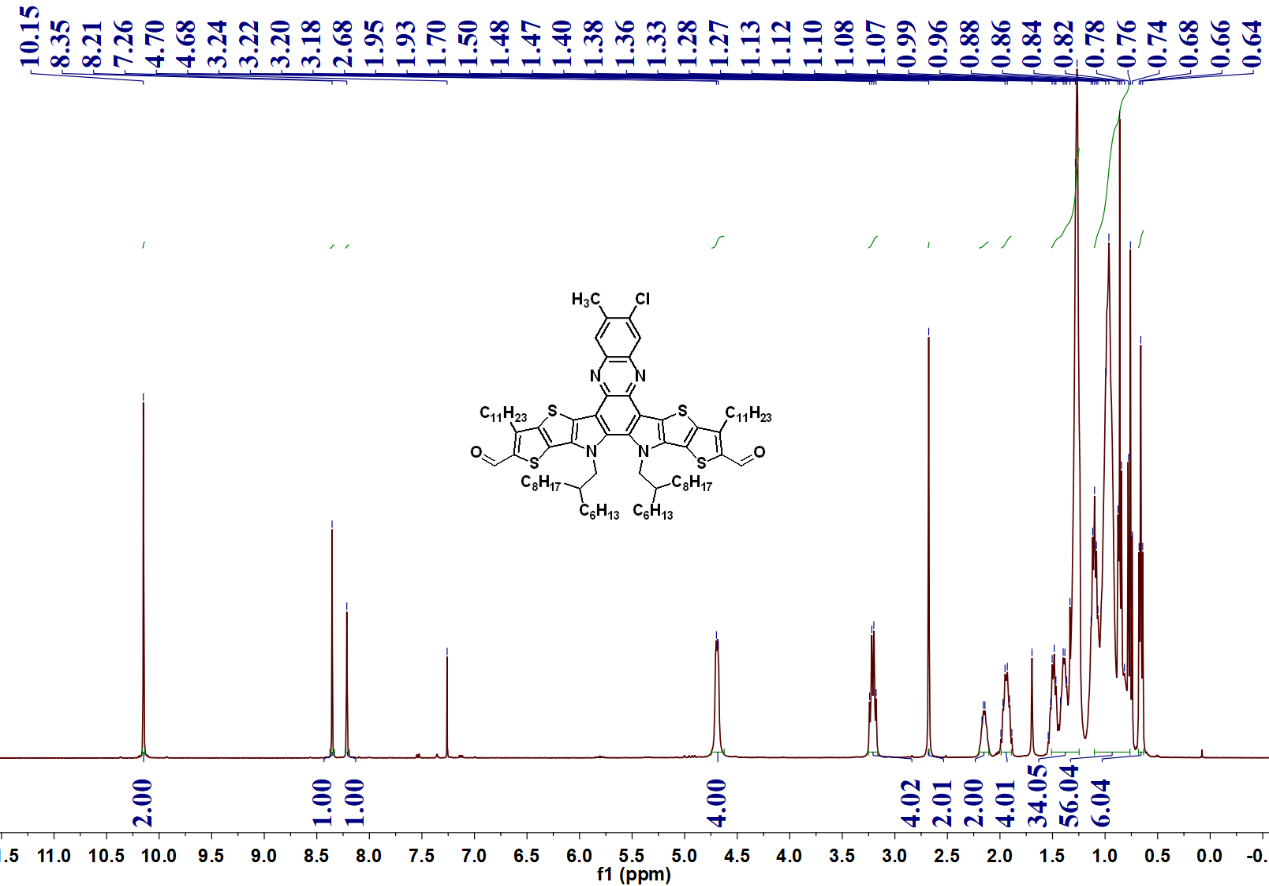


**Figure. S25. ^1^H NMR spectrum of 3b.**


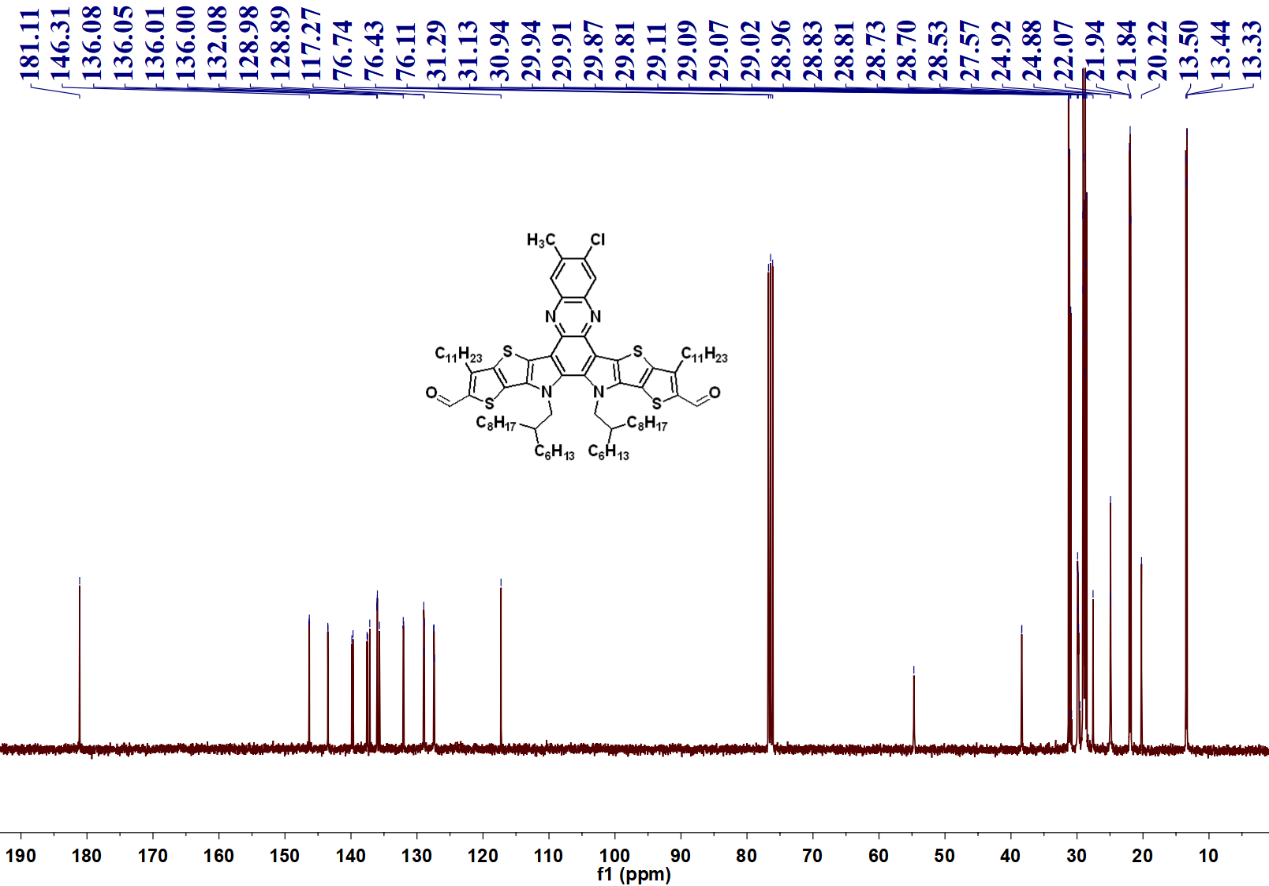


**Figure. S26. ^13^C NMR spectrum of 3b.**


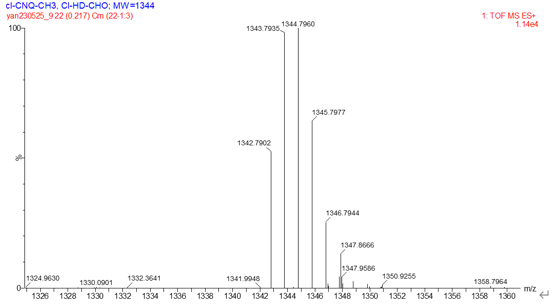


**Figure. S27. HRMS of 3b.**


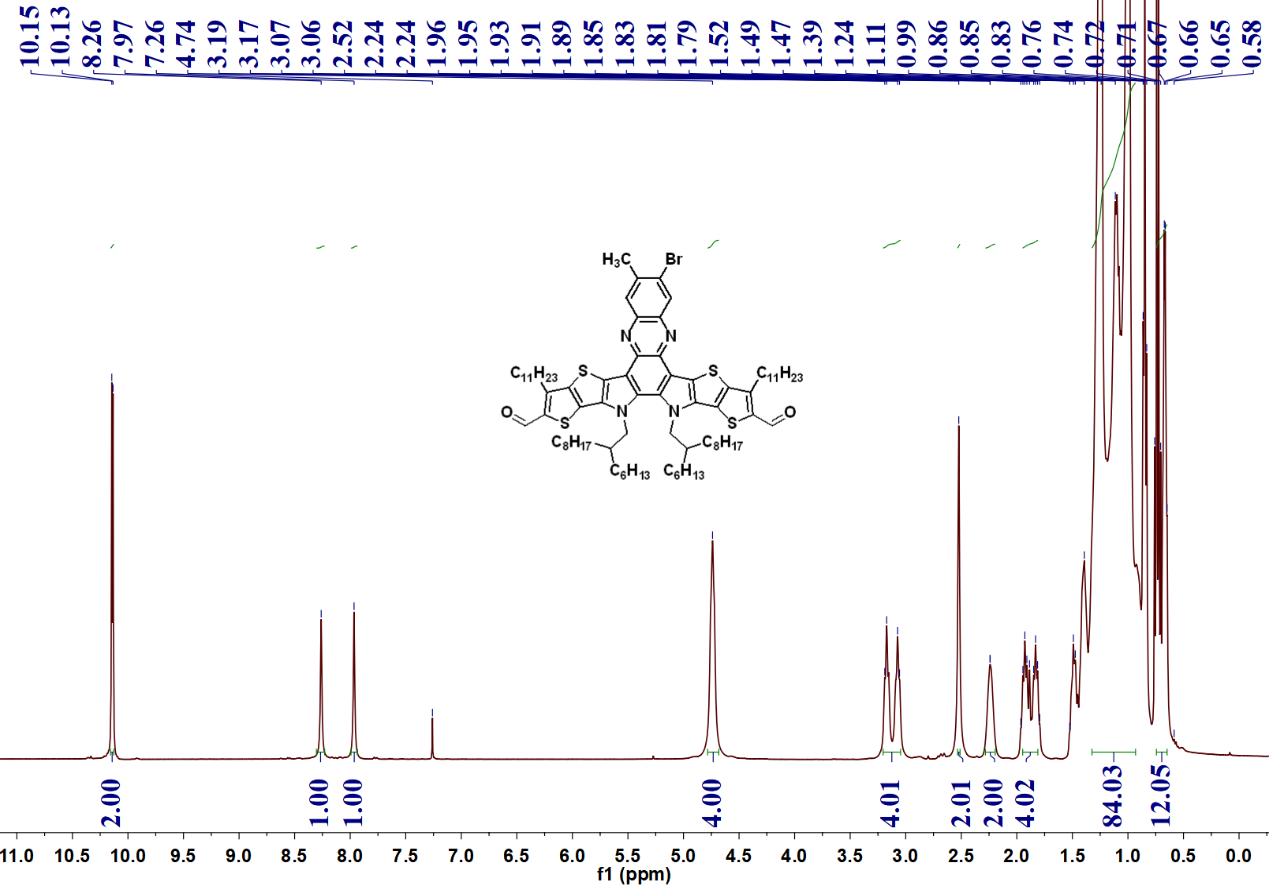


**Figure. S28. ^1^H NMR spectrum of 3c.**


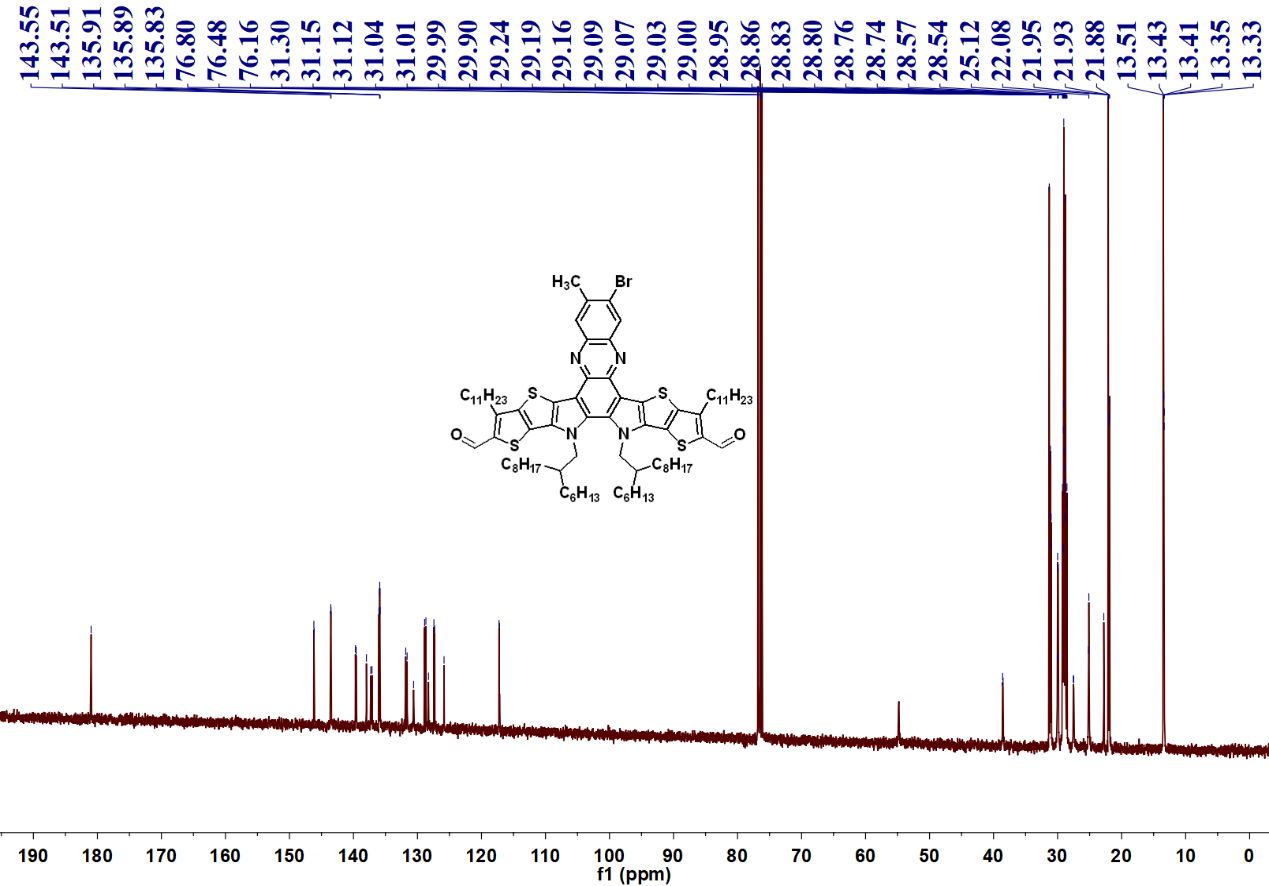


**Figure. S29. ^13^C NMR spectrum of 3c.**


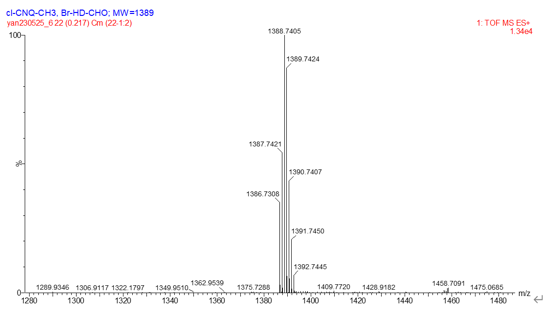


**Figure. S30. HRMS of 3c.**


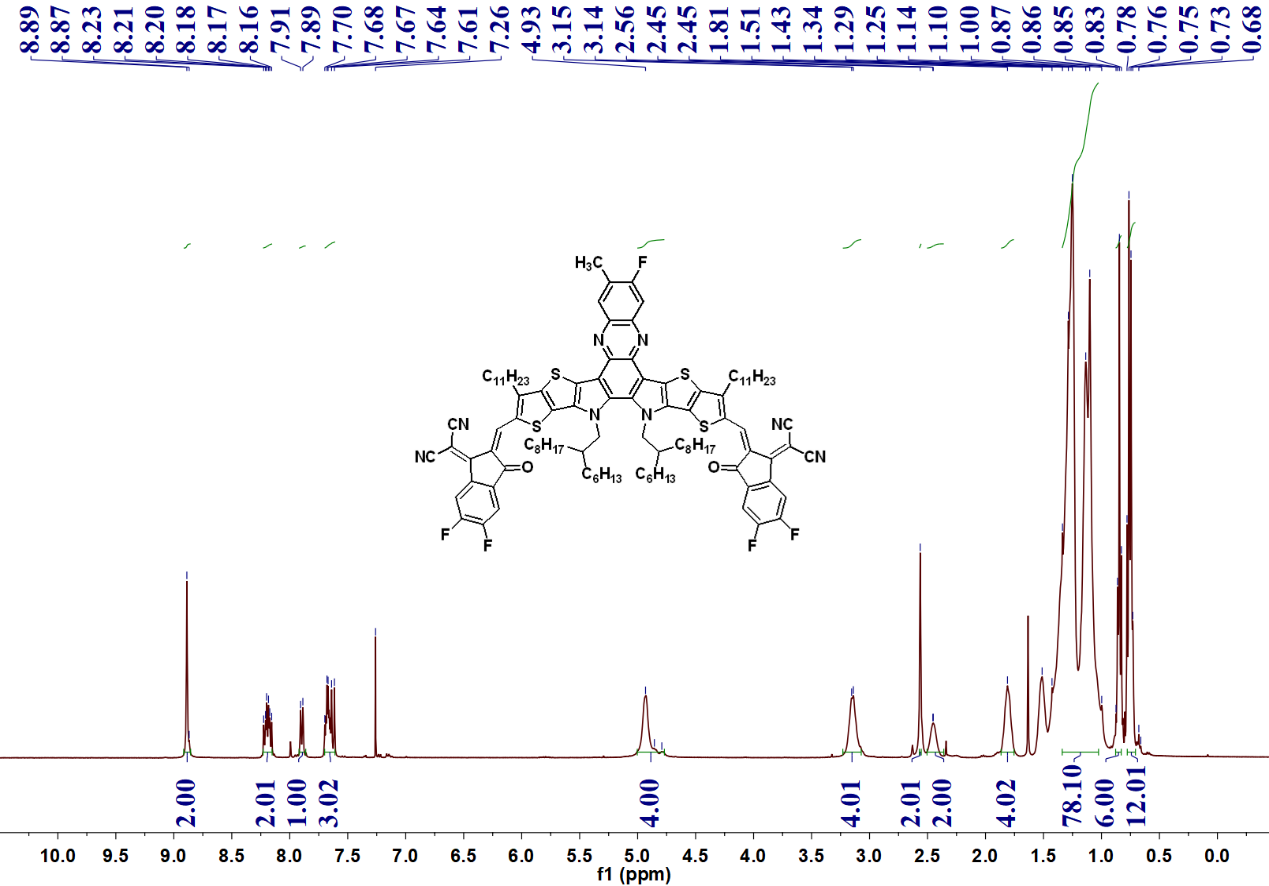


**Figure. S31. ^1^H NMR spectrum of BQx-MeF.**


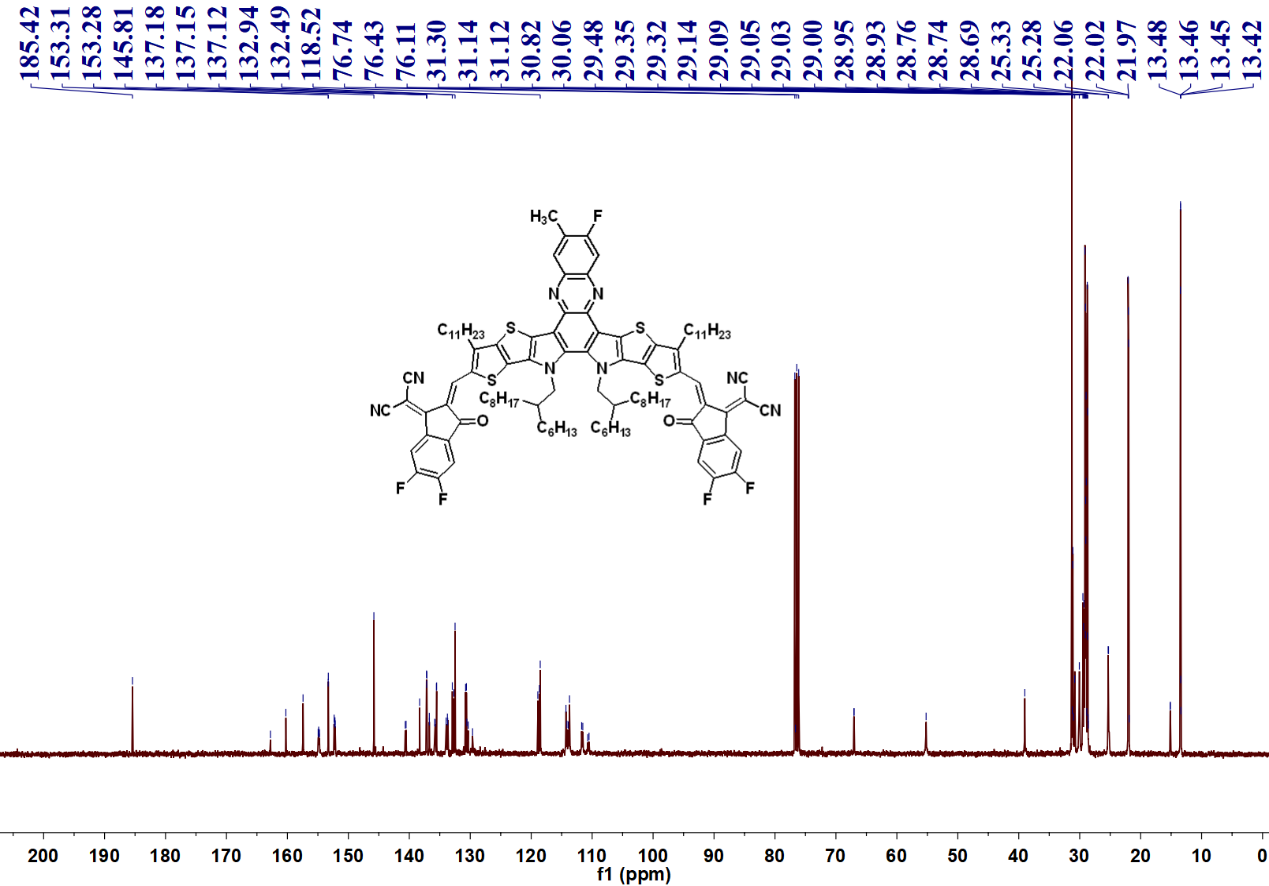


**Figure. S32. ^13^C NMR spectrum of BQx-MeF.**


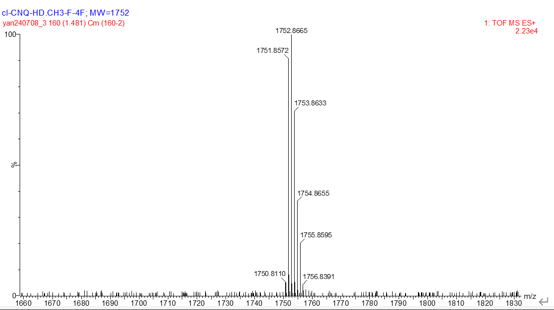


**Figure. S33. HRMS of BQx-MeF.**


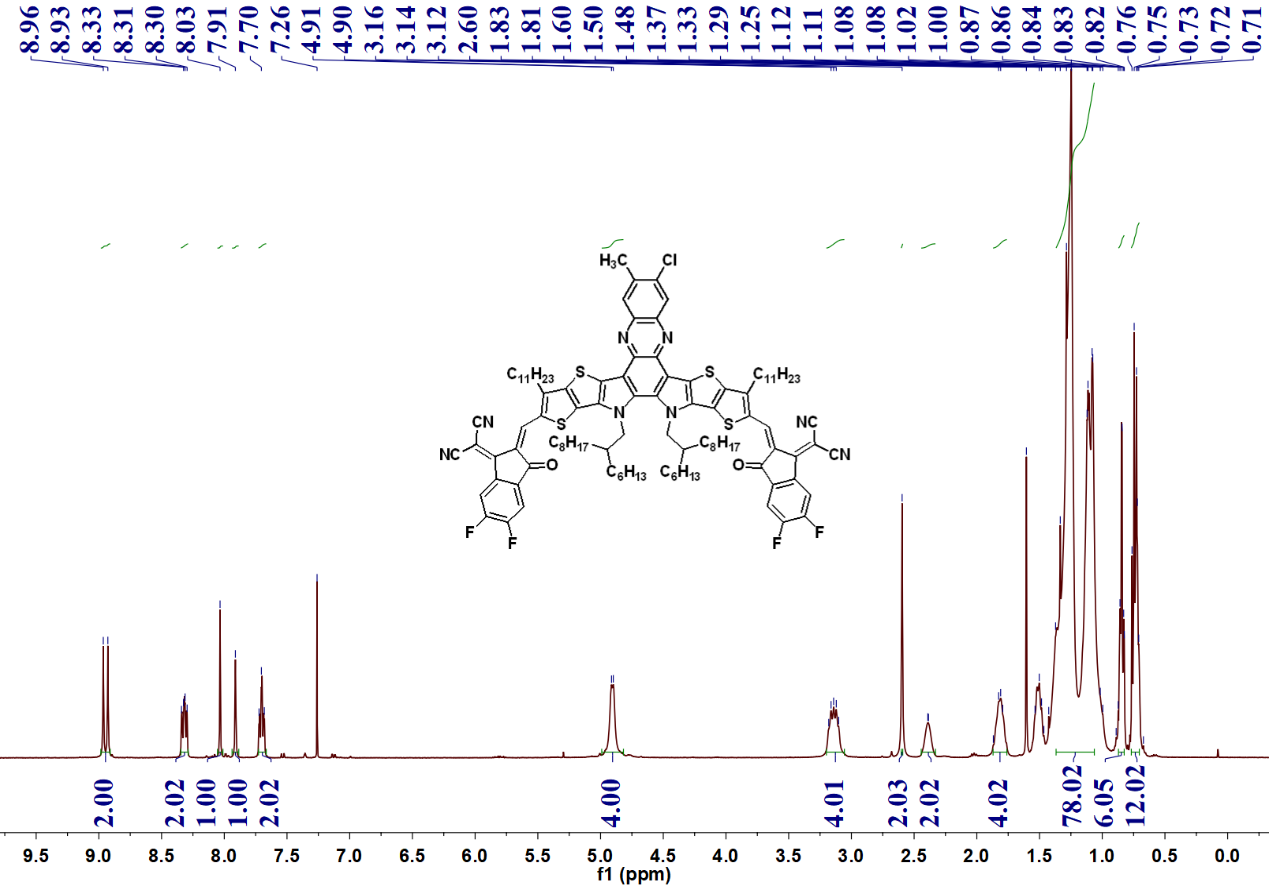


**Figure. S34. ^1^H NMR spectrum of BQx-MeCl.**


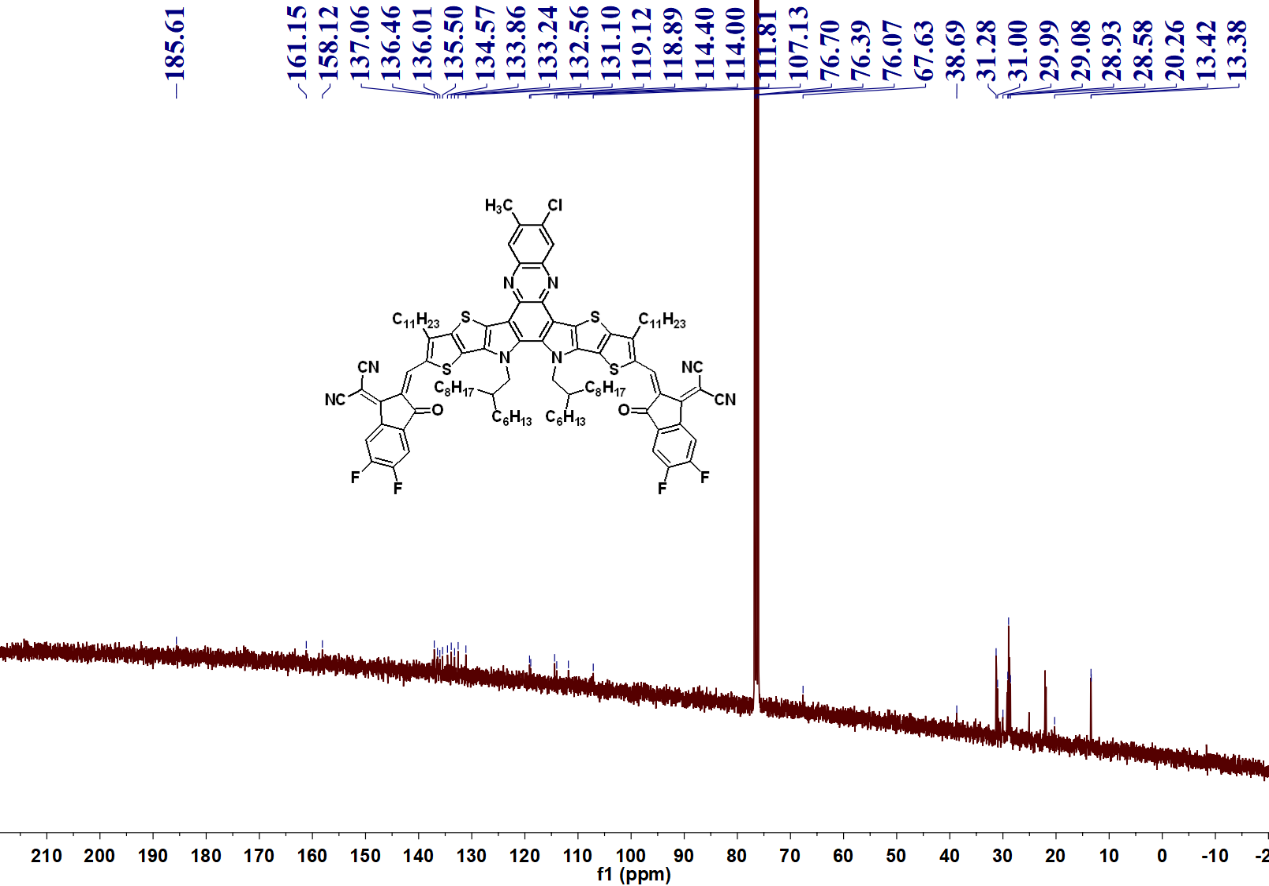


**Figure. S35. ^13^C NMR spectrum of BQx-MeCl.**


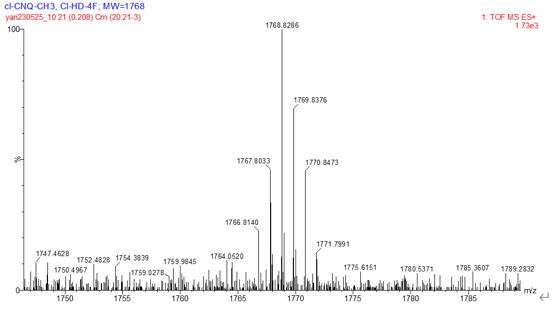


**Figure. S36. HRMS of BQx-MeCl.**


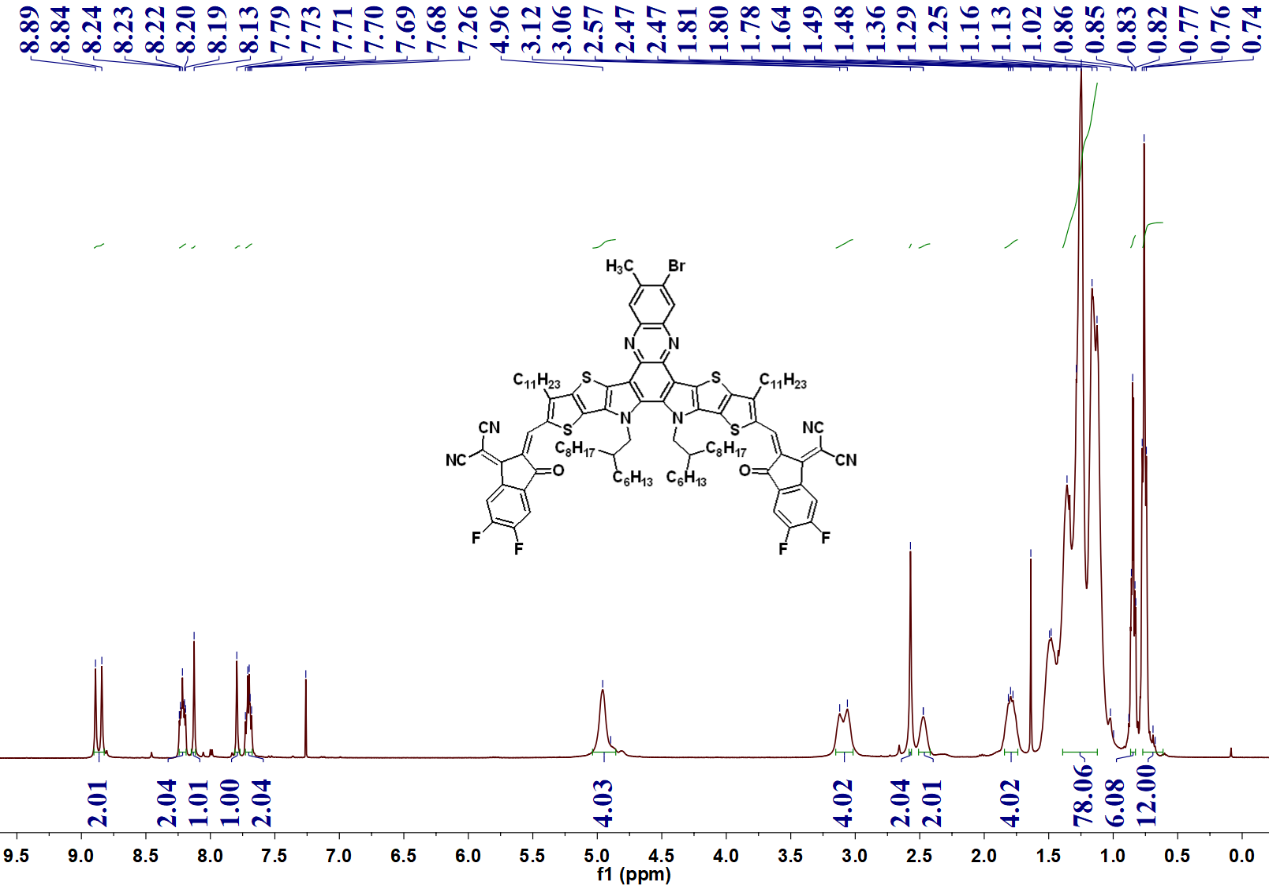


**Figure. S37. ^1^H NMR spectrum of BQx-MeBr.**


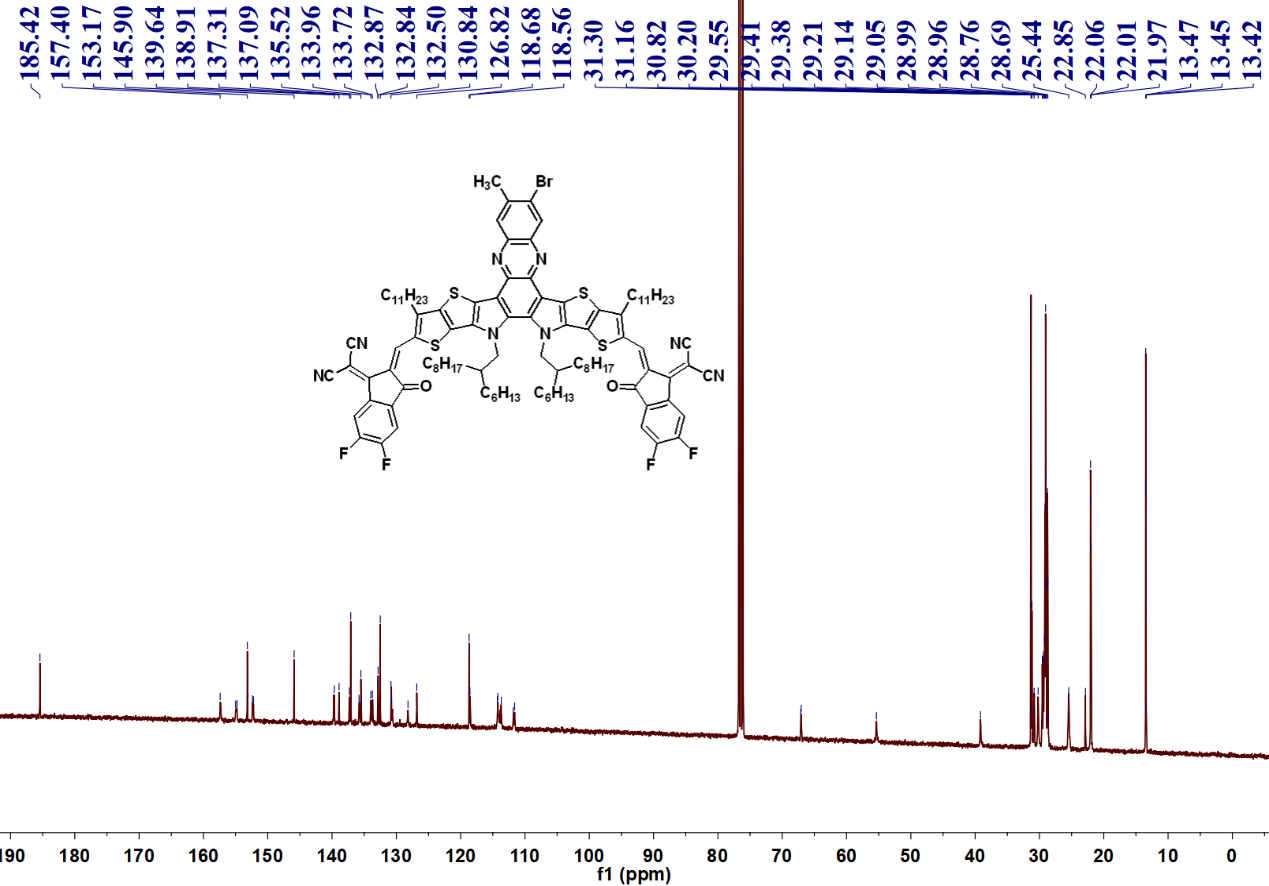


**Figure. S38. ^13^C NMR spectrum of BQx-MeBr.**


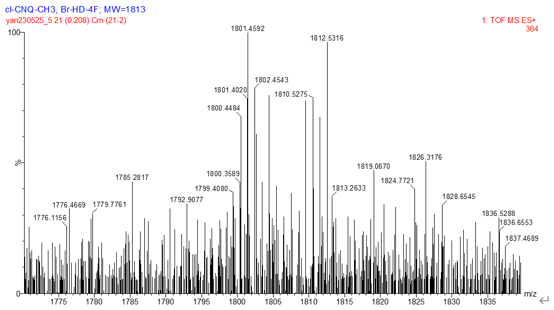


**Figure. S39. HRMS of BQx-MeBr.**

**4. Supplementary Tables S1-S8.**

**Table S1**. The optical and electrochemical parameters of SMAs in this work.

| **SMAs** | ***T*_d_ *^a^***  **(°C)** | ***λ*_max,sol_ *^b^***  **(nm)** | ***λ*_max,film_ *^c^***  **(nm)** | ***λ*_onset_*^e^***  **(nm)** | ***E*_g_^opt^ *^f^***  **(eV)** | ***E*_HOMO_ *^g^***  **(eV)** | ***E*_LUMO_ *^g^***  **(eV)** | **Solubility (mg/ml) *^h^*** |
| --- | --- | --- | --- | --- | --- | --- | --- | --- |
| **BQx-MeF** | 323.8 | 756 | 819 | 907 | 1.36 | -5.64 | -3.58 | 51 |
| **BQx-MeCl** | 325.6 | 754 | 818 | 904 | 1.37 | -5.69 | -3.60 | 58 |
| **BQx-MeBr** | 325.3 | 751 | 808 | 894 | 1.39 | -5.71 | -3.62 | 64 |

*^a^* The 5% weight-loss temperatures measured by TGA. Melting temperature measured by DSC. *^b^* Absorption maximum peak of solution. *^c^* Absorption maximum peak of thin film. *^d^* Molar extinction coefficient in solution. *^e^* Absorption onset of the *λ*_max,film_. *^f^* Optical bandgap estimated from the absorption onset of the thin film: *E*_g_^opt^ = 1240/*λ*_onset_. *^g^* Calculated according to the equation: *E*_HOMO/LUMO_ = -e(*E*_ox_/_red_ + 4.80) (eV). *^h^* chloroform as the solvent to dissolve the three acceptor materials.

**Table S2.** Summary of device parameters based different RPM of OSCs.*^a^*

| **Active layer** | ***V*_OC_ (V)** | ***J*_SC_ (mA cm^-2^)** | **FF (%)** | **PCE (%)** | **RPM** |
| --- | --- | --- | --- | --- | --- |
| PM6:**BQx-MeF** | 0.878 | 28.32 | 0.749 | 18.6 | 2400 |
|  | 0.878 | 28.21 | 0.761 | 18.8 | 2700 |
|  | 0.883 | 27.26 | 0.771 | 18.6 | 3000 |
| PM6:**BQx-MeCl** | 0.877 | 28.24 | 0.755 | 18.7 | 2300 |
|  | 0.876 | 28.71 | 0.761 | 19.1 | 2700 |
|  | 0.877 | 28.15 | 0.763 | 18.8 | 3000 |
| PM6:**BQx-MeBr** | 0.871 | 27.44 | 0.751 | 17.9 | 2400 |
|  | 0.872 | 28.01 | 0.753 | 18.4 | 2700 |
|  | 0.874 | 27.52 | 0.760 | 18.3 | 3000 |

*^a^* The standard deviations are based on measurements of 8 independent devices.

**Table S3**. Detailed *E*_loss_ parameters of the OSCs based on BQx-MeF, BQx-MeCl and BQx-MeBr.

| Active layer | *E*_g_*^a^*  (eV) | *V*_oc_ (V) | *E*_loss_  (eV) | *V*_SQ_*^b^* _OC_  _(V)_ | ∆*E*_1_  (eV) | *V*_rad_*^c^* _OC_  _(V)_ | ∆*E*_2_ *^d^*  (eV) | *∆E*_3_  (eV) | EQE_EL_ | PLQY (%) |
| --- | --- | --- | --- | --- | --- | --- | --- | --- | --- | --- |
| PM6:**BQx-MeF** | 1.394 | 0.880 | 0.515 | 1.135 | 0.261 | 1.086 | 0.049 | 0.205 | 3.6×10^-4^ | 4.5 |
| PM6:**BQx-MeCl** | 1.396 | 0.887 | 0.508 | 1.135 | 0.260 | 1.087 | 0.048 | 0.200 | 4.3×10^-4^ | 6.3 |
| PM6:**BQx-MeBr** | 1.404 | 0.876 | 0.528 | 1.142 | 0.262 | 1.088 | 0.055 | 0.211 | 2.8×10^-4^ | 3.7 |

*^a^* *E*_g_: determined from the derivatives of the EQE curve and the mean peak energy. *^b^* *V*_SQ OC_: Schokley-Queisser limit to *V*_OC_. *^c^* *V*_rad O_: radiative limit to *V*_OC_, measured using EQE_EL_. *^d^* ∆*E*_2_: voltage losses due to non-ideal absorption (it was calculated from EL and FTPS measurements). *^e^* ∆*E*_3_: voltage losses due to non-radiative recombination only.

**Table S4**. Charge mobility parameters of the optimized PM6: BQx-MeF, PM6: BQx-MeCl and PM6: BQx-MeBr devices.

| Device | *μ*_h_  (cm^2^ V^-1^s^-1^) | *μ*_e_  (cm^2^ V^-1^s^-1^) | *μ*_h_*_/_μ*_e_ | n_id,d_ | n_id,l_ | S |
| --- | --- | --- | --- | --- | --- | --- |
| PM6:**BQx-MeF** | 3.80 × 10^-4^ | 3.52 × 10^-4^ | 1.08 | 1.244 | 1.029 | 0.9767 |
| PM6:**BQx-MeCl** | 4.17 × 10^-4^ | 3.92 × 10^-4^ | 1.06 | 1.207 | 1.024 | 0.9775 |
| PM6:**BQx-MeBr** | 3.63 × 10^-4^ | 3.06 × 10^-4^ | 1.19 | 1.288 | 1.133 | 0.9702 |

**Table S5**. Morphology parameters extracted from the GIWAXS measurements.

| Film | q  (010, Å^-1^) | d-space *^a^*  (010, Å) | CCL *^b^* (010, Å) | FWHA  (010, Å^-1^) | q  (100, Å-1) | d-space *^a^* (100, Å) | CCL *^b^* (100, Å) | FWHA  (100, Å^-1^) |
| --- | --- | --- | --- | --- | --- | --- | --- | --- |
| **BQx-MeF** | 1.72 | 3.65 | 31.40 | 0.180 | 0.30 | 21.22 | 89.71 | 0.063 |
| **BQx-MeCl** | 1.73 | 3.64 | 32.71 | 0.172 | 0.30 | 21.14 | 95.15 | 0.059 |
| **BQx-MeBr** | 1.72 | 3.65 | 28.55 | 0.198 | 0.30 | 21.22 | 87.22 | 0.065 |
| **PM6: BQx-MeF** | 1.72 | 3.66 | 26.17 | 0.216 | 0.29 | 21.69 | 86.55 | 0.065 |
| **PM6: BQx-MeCl** | 1.72 | 3.65 | 33.05 | 0.171 | 0.29 | 21.66 | 89.71 | 0.063 |
| **PM6: BQx-MeBr** | 1.72 | 3.66 | 24.15 | 0.234 | 0.29 | 21.71 | 84.32 | 0.067 |

*^a^* Calculated from the equation: d-spacing = 2π/q. *^b^* Obtained from the Scherrer equation: CCL = 2πK/FWHM, where FWHM is the full-width at half-maximum and K is a shape factor (K = 0.9 here).

**Table S6.** The fitted parameter for hole transfer kinetics. Decomposed time components from **Figure. 5g** were fitted with biexponential function, with the fast (*τ*_1_) component attributed to exciton dissociation and slow (*τ*_2_) component attributed to exciton diffusion.

| **Sample** | ***τ*_1_, ps** | ***τ*_2_, ps** |
| --- | --- | --- |
| **PM6:BQx-MeF** | 3.96 ±0.80 | 28.50 ±1.7 |
| **PM6:BQx-MeCl** | 1.39 ±0.02 | 25.30 ±1.5 |
| **PM6:BQx-MeBr** | 1.49 ±0.03 | 19.64 ±0.8 |

**Table S7.** The fitted parameter for local exciton kinetics. (**Figure. 5h)**

| **Sample** | ***τ*_1_, ps** | ***τ*_2_, ps** |
| --- | --- | --- |
| **PM6:BQx-MeF** | 0.87 ±0.11 | 22.5 ±2.2 |
| **PM6:BQx-MeCl** | 1.29 ±0.05 | 30.7 ±2.5 |
| **PM6:BQx-MeBr** | 1.36 ±0.06 | 37.1 ±1.7 |

**Table S8.** The fitted parameter for delocalized state kinetics of pure acceptor. (**Figure. 5i)**

| Sample | *τ*_rise_, ps | *τ*_1_, ps | *τ*_2_, ps |
| --- | --- | --- | --- |
| **BQx-MeF** | 0.16 ±0.01 | 7.33 ±0.45 | 82.64 ±3.3 |
| **BQx-MeCl** | 0.13 ±0.01 | 7.74 ±0.65 | 80.60 ±3.5 |
| **BQx-MeBr** | 0.18 ±0.02 | 8.48 ±0.55 | 90.90 ±3.7 |

**5. Reference.**

[1] B. Hess, C. Kutzner, D. van der Spoel, E. Lindahl, *J Chem Theory Comput*, **2008**, *4*, 435-447;

[2] S. Pronk, S. Páll, R. Schulz, P. Larsson, P. Bjelkmar, R. Apostolov, M. R. Shirts, J. C. Smith, P. M. Kasson, D. van der Spoel, B. Hess, E. Lindahl, *Bioinformatics*, **2013**, *29*, 845-854.

[3] J. Wang, R. M. Wolf, J. W. Caldwell, P. A. Kollman, D. A. Case, *J Comput Chem*, **2004**, *25*, 1157-1174.

[4] T. Lu, http://sobereva.com/soft/Sobtop., *June***, 2023**.

[5] T. Lu, F. Chen, *J Comput Chem*, **2012**, *33*, 580-592.

[6] H. Chen, H. Liang, Z. Guo, Y. Zhu, Z. Zhang, Z. Li, X. Cao, H. Wang, W. Feng, Y. Zou, L. Meng, X. Xu, B. Kan, C. Li, Z. Yao, X. Wan, Z. Ma, Y. Chen, *Angew Chem Int Ed.*, **2022**, *61*, e202209580.

[7] L. Chen, C. Zhao, H. Yu, A. Sergeev, L. Zhu, K. Ding, Y. Fu, H. Ng, C. Kwok, X. Zou, J. Yi, X. Lu, K. Wong, H. Ade, G. Zhang, H. Yan, *Adv. Energy Mater*., **2024**, 2400285.
